# Dynamics of early electroencephalographic patterns and epileptic seizures in acute intracerebral hemorrhage: A prospective controlled study

**DOI:** 10.1111/epi.70017

**Published:** 2025-11-18

**Authors:** Ziad Al‐Fatuhi‐Al‐Jundi, Salomé Avenas, Pierre Tankéré, Frédéric Philipeau, Pierre Garnier, Laure Mazzola, Nathalie Andre‐Obadia, Sébastien Boulogne, Hélène Catenoix, Sylvain Rheims, Tae‐Hee Cho, Julia Fontaine, Laura Mechtouff, Elodie Ong, Yves Berthezene, Anne Termoz, Nathalie Perreton, Julie Haesebaert, Muriel Rabilloud, Laurent Derex, Laure Peter‐Derex

**Affiliations:** ^1^ Department of Functional Neurology and Epileptology Neurological Hospital, University Hospital Lyon France; ^2^ Department of Biostatistics, Edouard Herriot Hospital Lyon University Hospital Lyon France; ^3^ Center for Sleep Medicine Croix‐Rousse Hospital, University Hospital Lyon France; ^4^ Stroke Unit, Department of Neurology Fleyriat Hospital Bourg en Bresse France; ^5^ Stroke Center, Department of Neurology Saint‐Etienne University Hospital Saint‐Etienne France; ^6^ Clinical Neurophysiology Unit, Department of Neurology Saint‐Etienne University Hospital Saint‐Etienne France; ^7^ Lyon Neuroscience Research Center, Centre National de la Rcherche Scientifique Unité Mixte de Recherche 5292, Insitut National de la Santé Et de la Recherche Médicale U1028 Lyon France; ^8^ Stroke Center Neurological Hospital, University Hospital Lyon France; ^9^ Department of Neuroradiology Neurological Hospital, University Hospital Lyon France; ^10^ Public Health Unit, Clinical Research and Epidemiology Department Lyon University Hospital Lyon France; ^11^ Research on Health Care Performance RESHAPE, Insitut National de la Santé Et de la Recherche Médicale U1290, Université Claude Bernard Lyon 1 Lyon France; ^12^ Biometry and Evolutionary Biology Laboratory, Centre National de la Rcherche Scientifique Unité Mixte de Recherche, Biostatistics Health Team Villeurbanne France

**Keywords:** acute symptomatic seizures, continuous electroencephalography, interictal epileptiform discharges, intracerebral hemorrhage, rhythmic and periodic patterns

## Abstract

**Objective:**

Acute symptomatic seizures (ASyS) occur in up to 30% of patients with intracerebral hemorrhage (ICH) when continuous electroencephalography (cEEG) is used, potentially worsening outcomes. Identification of early EEG biomarkers of ASyS may help guide personalized antiseizure medication (ASM) prophylaxis. Here, we aimed to describe early interictal EEG patterns, their dynamics, and their association with seizure risk, considering the effect of prophylactic levetiracetam.

**Methods:**

This prospective analysis used data from the PEACH phase 3 trial (2017–2020), which enrolled adults with acute spontaneous supratentorial ICH, randomized to receive levetiracetam or placebo. Patients underwent systematic 48‐h cEEG within 48 h of symptom onset. Electrographic seizures and interictal EEG patterns were analyzed using standardized terminology of the American Clinical Neurophysiology Society. Associations between rhythmic and periodic patterns (RPPs) and seizures with clinical and radiological variables were assessed using univariate analyses. We also conducted exploratory testing of the CAV (cortical involvement, age < 65 years, volume > 10 mL) score for predicting ASyS, incorporating RPPs and ASM exposure.

**Results:**

Forty‐two patients were included (median [Q1–Q3] age = 72 [60–79] years, 29% women), 19 in the levetiracetam group. Interictal EEG abnormalities were common and not influenced by ASM, including background asymmetry (73%), sporadic epileptiform discharges (62%), and RPPs (52%). RPPs were associated with ICH volume (*p* = .039) and cortical involvement (*p* = .003). Among patients with RPPs, 50% developed ASyS (20% in those treated with ASM vs. 75% in untreated patients, *p* = .030). Most patients (91.7%) with seizures had RPPs that preceded seizures, in >90% cases by 12 (Q1–Q3 = 4–25) h. Integrating RPPs into the CAV model led to an improvement of ASyS prediction (area under the curve = .949 vs. .918, *p* = .53) that was statistically nonsignificant.

**Significance:**

RPPs are strong markers of ictogenesis in acute ICH and precede ASyS, thus offering a potential therapeutic window. These findings support the use of early cEEG for risk stratification and personalized ASM prophylaxis.

**Key points**

- cEEG detects RPPs in approximately 50% of patients with acute ICH.
- RPPs are associated with ICH volume and cortical involvement but are not influenced by ASM.
- Half of the patients with RPPs experience seizures (20% in those treated with ASM vs. 75% in untreated patients).
- When present, RPPs precede seizures in >90% of cases.
- The CAV model including ASM shows good predictive performance for predicting ASyS, and the addition of RPPs may offer a modest, yet nonsignificant, improvement.

## 1 INTRODUCTION

Spontaneous intracerebral hemorrhage (ICH) accounts for 10%–15% of all incident strokes.^1^ Although less frequent than ischemic strokes, ICH carries a significant burden due to its poor prognosis.^2^, ^3^ The mortality rate is approximately 36% at 1 month,^4^ and only 16.7%–24.6% of patients achieve functional independence after 1 year.^5^ In contrast to ischemic strokes, effective acute treatments for ICH remain limited,^2^ highlighting the urgent need for new approaches to improve outcomes in ICH. One such potential strategy is the prevention of early epileptic seizures.

A recent meta‐analysis estimated the overall incidence of epileptic seizures in spontaneous ICH at 9.5%.^6^ When focusing only on acute symptomatic seizures (ASyS), that is, seizures occurring within 7 days of stroke,^7^ the reported incidence ranges from 5% to 14%.^8^, ^9^, ^10^, ^11^ Notably, this prevalence has increased over the past years.^12^ However, most studies on seizures in ICH rely on clinical observation, failing to detect many seizures, particularly those without observable symptoms in patients with severe deficits or impaired consciousness.^13^ When continuous electroencephalographic (cEEG) monitoring is used, seizure incidence increases significantly, reaching up to 30%.^14^, ^15^, ^16^ Some studies have suggested that early seizures might be associated with hematoma or edema expansion,^14^ poorer functional outcomes, and higher mortality rates,^12^, ^17^, ^18^, ^19^ especially in cases of status epilepticus.^10^ Nevertheless, findings remain inconsistent across studies,^8^, ^20^, ^21^, ^22^ possibly due to confounding factors affecting both prognosis and seizure risk, or to detection bias arising from the reliance on clinical monitoring in many studies, which overlooks subclinical seizures.

We recently conducted a randomized study (PEACH trial) suggesting that antiseizure medications (ASMs) may be safe and effective to prevent ASyS in acute ICH.^16^ Identifying patients at high risk for ASyS is therefore essential to enable personalized treatment strategies. Several clinical, demographic, and radiological factors have been associated with the risk of ASyS, including hematoma volume, cortical involvement, lobar localization, alcohol use, and younger age, possibly because subclinical seizures might be particularly underestimated in the elderly.^6^, ^8^, ^23^, ^24^, ^25^, ^26^ In line with the CAVE score (cortical involvement, age < 65 years, ICH volume > 10 mL, and presence of early seizures^27^), which was developed to predict the risk of late seizures, Bunney et al. proposed the CAV score to predict early seizures, demonstrating good performance (area under the curve [AUC] = .72, 95% confidence interval = .62–.82).^26^ In addition, neurophysiological parameters appear promising. Studies using cEEG in ICH patients have highlighted specific electrographic patterns associated with increased seizure risk. Pioneering works by Claassen et al.^15^ found that periodic epileptiform discharges, periodic lateralized epileptiform discharges, and stimulus‐induced rhythmic, periodic, or ictal discharges (as defined in Claassen et al.^9^, ^28^) are associated with seizures in ICH. The predictive value of periodic and rhythmic patterns, including lateralized periodic discharges (LPDs) and lateralized rhythmic delta activity (LRDA), were confirmed in recent works.^29^, ^30^ In addition to LPDs and LRDA, data from large populations of critically ill patients (including ICH) identified other EEG patterns associated with seizure risk such as the presence of bilateral independent periodic discharges, generalized periodic discharges (GPDs), and the characteristics of LPDs, GPDs, or LRDA including higher frequency, association with “plus features,” or high prevalence (for LPDs and GPDs).^31^, ^32^ These electrophysiological markers seem particularly relevant as they are noninvasive and cost‐effective. Moreover, some of these patterns may independently contribute to cerebral damage,^33^ raising the question of whether they should be treated even in the absence of clinical seizures. However, most studies on EEG patterns in ICH are retrospective, often including patients monitored with cEEG due to clinical deterioration or presumed seizure risk, with some already receiving ASM. Additionally, there is substantial heterogeneity in the timing of EEG recordings relative to the onset of ICH.

In the PEACH trial, cEEG monitoring was systematically conducted for 48 h during the acute phase of ICH^16^; this provides a unique opportunity to study the characteristics of EEG patterns without the bias introduced by clinician‐driven decisions regarding cEEG indication of ASM use. In the present work, we aimed at (1) describing interictal EEG patterns, their prevalence, and their temporal dynamics in acute ICH, in patients with versus without ASyS, and in those with versus without ASM prophylaxis; and (2) investigating the determinants of such patterns and their association with ASyS risk.

## 2 MATERIALS AND METHODS

### 2.1 Participants

Patients were enrolled as part of the PEACH trial, conducted from June 2017 to April 2020 across the stroke units of Lyon University Hospital, Saint‐Etienne University Hospital, and Bourg‐en‐Bresse Hospital. Inclusion criteria were as follows: age ≥ 18 years, spontaneous supratentorial ICH, and admission within 24 h of symptom onset. Exclusion criteria are detailed in Peter‐Derex et al.^16^ and included the following: National Institutes of Health Stroke Scale (NIHSS) score > 25; intracerebral hemorrhage secondary to trauma, vascular malformation, hemorrhagic transformation of ischemic stroke, or tumor; and current use of ASM or history of epilepsy.

### 2.2 PEACH study design

PEACH was a parallel‐group, double‐blinded, randomized (1:1), placebo‐controlled, phase 3 trial aiming to demonstrate that prophylactic treatment with levetiracetam would reduce the risk of early seizures in acute ICH. The detailed protocol is available in Peter‐Derex et al.^16^ Briefly, the treatment with levetiracetam (or placebo) was administered at full dose (1 g per day) for 30 days before gradual tapering, with an intravenous initiation within 24 h of randomization and then an oral administration when swallowing was possible. cEEG monitoring was initiated as early as possible within 24 h of randomization (i.e., within 48 h of symptom onset) and following treatment initiation. The EEG was conducted at the patient's bedside and digitally recorded using 11 electrodes positioned on the scalp according to the international 10–20 system: Fp2, C4, T4, O2, Fp1, C3, T3, O1, Fz, Cz, and Pz, along with an electrocardiographic lead. A Holter device (Morpheus, MICROMED) was used, ensuring that recordings were not accessible in real time and remained blinded to investigators. This approach prevented any influence of EEG findings on ASM treatment decisions. Notably, as cEEG is not routinely performed in stroke units, this protocol did not disadvantage patients. Brain imaging (computed tomography or magnetic resonance imaging) was performed at baseline and 72 h.

### 2.3 Data collection

#### 2.3.1 Clinical data

Clinical information gathered for the present study included the following: age; sex; history of hypertension, ischemic, or hemorrhage stroke, anticoagulant or antiaggregant use, smoking, renal failure, diabetes, cardiac ischemia, alcohol use disorder, pre‐ICH modified Rankin score; and clinical seizures at 72 h and 1 month.

#### 2.3.2 Imaging data

Brain imaging was read centrally by neuroradiologists masked to group assignments. Radiological outcomes included the following: ICH volume measured at baseline and 72 h using semiautomated computerized planimetry; deep or lobar localization; cortical involvement; topography; hemorrhage side; thalamic involvement; presence of intraventricular hemorrhage, subarachnoid hemorrhage, or hydrocephalus; and midline shift initially and at 72 h.

#### 2.3.3 Electrophysiological data

Initial analysis of EEG recordings in the context of the PEACH trial was conducted offline by a panel of electrophysiology experts to identify electrographic seizures. For the present study, raw data were rereviewed. Recordings were first both analyzed (masked to group assignments and imaging data) by Z.A.‐F.‐A.‐J. and then reviewed by L.P.‐D., both being board certified neurophysiologists. The analysis followed the American Clinical Neurophysiology Society (ACNS) Standardized Critical Care EEG Terminology,^34^ including the description of background rhythm, sporadic epileptiform discharges, rhythmic and periodic patterns (RPPs) consisting of periodic discharges (PDs) and rhythmic delta activity (RDA), and electrographic seizure (ESz; details are provided in Table S1).

### 2.4 Statistical analysis

Clinical, radiological, and EEG variables are described by the median (Q1–Q3) for quantitative data. Qualitative variables are described by number of patients, number of missing values, frequency, and percentage of each modality. We describe the time between the onset of ICH symptoms, the first occurrence of an RPP, and in patients with ESz, the first seizure.

Univariate analyses were performed to assess the association between various clinical, radiological, and electroencephalographic factors and the occurrence of RPPs or ESz. For categorical variables, the chi‐squared test was applied when its assumptions were met; otherwise, Fisher exact test was used. For continuous variables, comparisons were conducted using the Wilcoxon rank‐sum test. To control the familywise error rate, Holm's step‐down method was applied for multiple comparisons, with a significance threshold set at α ≤ .05. Exploratory analysis was conducted to test the CAV score in our population and investigate the added value of RPPs for ESz prediction. We used a multivariate logistic regression with Wald tests to determine whether a predictor variable was significant. receiver operating characteristic curves were generated to assess the performance of the logistic regression models evaluated. We compared the predictive performance using the operating characteristic curves (AUC) of two logistic regression models, one including the RPP variable and one without. The statistical significance of the difference between the two correlated AUCs was assessed using DeLong test. All statistical analyses were performed using R‐4.4.1.

### 2.5 Ethical considerations

The PEACH trial was approved by the institutional review board (CPP Sud‐Est, No. 2014–000436–41), the National Drug Safety Agency (No. 141303A‐31), and the National Commission for Data Protection (No. DR‐2015–539). Written informed consent was obtained from participants or, when they were unable, from a proxy (relative or legal representative), with participant confirmation sought at the earliest opportunity. The study oversight was conducted by the Clinical Research Unit of Lyon University Hospital (Lyon, France).

## 3 RESULTS

### 3.1 Characteristics of the patients

Forty‐two patients (median [Q1–Q3] age = 72 [60–79] years, 29% women) were included, 19 in the levetiracetam group and 23 in the placebo group (flowchart is provided in Figure S1). Clinical and radiological characteristics of patients are presented in Table 1. The median volume of hematoma was 14 (Q1–Q3 = 5–24) mL. Twelve patients (29%) had lobar hematoma localization, and 53.7% had cortical involvement, with some deep hematoma spreading to the insular or temporal cortex. Intracerebral hematoma was associated with intraventricular hemorrhage in 24% patients and with subarachnoid hemorrhage in 17% of patients. The median midline shift at baseline was 3 (Q1–Q3 = 0–4) cm. The median increase in midline shift at 72 h was 1 (Q1–Q3 = 0–3) cm.

**TABLE 1:** Clinical and imaging characteristics of the population.

Characteristic	Treatment		Acute symptomatic seizures^a^		Overall, *N* = 42^b^
	Levetiracetam, *n* = 19^b^	Placebo, *n* = 23^b^	Absent, *n* = 29^b^	Present, *n* = 13^b^	
Clinical characteristics					
Age, years	77 [71–81]	63 [52–76]	73 [60.5–79.5]	64 [55.5–80]	72 [60–79]
Sex (% female)	7 (36.8%)	5 (21.7%)	10 (34.5%)	2 (15.4%)	12 (29%)
Medical history					
Hypertension	13/17 (76%)	12/21 (57.1%)	18/27 (66.7%)	7/11 (63.6%)	25/38 (60%)
Ischemic stroke	3 (16%)	1 (4.3%)	4 (13.8%)	0 (0%)	4 (9.5%)
ICH	0 (0%)	1 (4.3%)	0 (0%)	1 (7.7%)	1 (2.4%)
Alcohol use disorder	0/17 (0%)	5 (22%)	1/27 (3.7%)	4 (30.8%)	5/40 (12%)
Use of anticoagulants	1 (5.3%)	2 (8.7%)	3 (10.3%)	0 (0%)	3 (7.1%)
Smoking	1 (5.3%)	3 (13%)	2 (6.9%)	2 (15.4%)	4 (9.5%)
Renal failure	2 (11%)	2 (8.7%)	3 (10.3%)	1 (7.7%)	4 (9.5%)
Diabetes	8 (42%)	1/22 (4.5%)	6/28 (21.4%)	3 (23.1%)	9/41 (22%)
Myocardial infarction	2 (11%)	1 (4.3%)	3 (10.3%)	0 (0%)	3 (7.1%)
Prestroke mRS					
0	13 (68.4%)	21 (91.3%)	22 (75.9%)	12 (92.3%)	34 (80.9%)
1	2 (10.5%)	1 (4.3%)	2 (6.9%)	1 (7.7%)	3 (7.1%)
2	3 (15.8%)	1 (4.3%)	4 (13.8%)	0 (0%)	4 (9.5%)
3	1 (5.3%)	0 (0%)	1 (3.4%)	0 (0%)	1 (2.4%)
Imaging characteristics					
Lobar localization	9 (47%)	3 (13%)	7 (24%)	5 (38%)	12 (29%)
Cortical involvement	9/18 (50%)	13 (56.5%)	10/28 (35.7%)	12 (92.3%)	22/41 (53.7%)
Hematoma volume, mL	8 [3–24]	15 [5–24]	5 [3–16]	27 [20–45]	14 [5–24]
Thalamic involvement	7 (37%)	9 (39%)	15 (52%)	1 (7.7%)	16 (38%)
Intraventricular extension	4 (21%)	6 (26%)	8 (28%)	2 (15%)	10 (24%)
Meningeal extension	4 (21%)	3 (13%)	2 (6.9%)	5 (38%)	7 (17%)
Midline shift at baseline, cm	3.0 [.0–4.0]	3.0 [.0–5.0]	2.0 [.0–4.0]	4.0 [3.0–5.0]	3.0 [.0–4.0]
*n*	11	15	17	9	26
Change in midline shift at 72 h, cm	1 [0–4.5]	1 [0–3]	0 [.0–1.0]	3 [2.0–5.0]	1 [.0–3.0]
*n*	9	16	16	9	25

*Note*: In the case of missing data, sample sizes are indicated as *n*.

Abbreviations: ICH, intracerebral hemorrhage; mRS, modified Rankin Score.

^**a**^ Acute symptomatic seizure refers to seizure related to ICH, within 72 h following inclusion, including clinical and electrographic seizures. In our cohort, all seizures were electrographic.

^b^ Median [Q1–Q3] or *n* (%).

A total of 13 patients experienced seizures within the first 72 h following inclusion: three in the levetiracetam group and 10 in the placebo group. All seizures (*n* = 164, six in the levetiracetam group and 158 in the placebo group) were electrographic. Additionally, one patient in the placebo group had a clinical seizure 12 days after randomization.

### 3.2 Electrophysiological features recorded during cEEG

#### 3.2.1 Interictal patterns

Most patients exhibited an asymmetric EEG background in terms of frequency (73%, and 100% among those with ESz) and amplitude (64.5%). Slower frequencies were typically observed ipsilateral to the hemorrhage (90.3%), whereas reduced amplitude was more often contralateral (74%). Reactivity was absent on at least one side in four patients (9.5%), all of whom experienced seizures. An abnormal anteroposterior gradient was found in 40% of patients, most frequently ipsilateral to the hemorrhage. Sporadic epileptiform discharges were observed in 62% patients, mostly on the same side as the hemorrhage (86%) and with prominent temporal topography (73%; Table 2).

**TABLE 2:** Description of electroencephalographic features according to the American Clinical Neurophysiology Society guidelines.

Electroencephalographic characteristics^a^	Treatment		Acute symptomatic seizures^b^		Overall, *N* = 42^c^
	Levetiracetam, *n* = 19^c^	Placebo, *n* = 23^c^	Absent, *n* = 29^c^	Present, *n* = 13^c^	
Symmetry in frequency					
Marked asymmetry	12 (63%)	10 (43%)	13 (45%)	9 (69%)	22 (52%)
Mild asymmetry	3 (16%)	6 (26%)	5 (17%)	4 (31%)	9 (21%)
Symmetry	3 (16%)	6 (26%)	9 (31%)	0 (0%)	9 (21%)
Unclear	1 (5.3%)	1 (4.3%)	2 (6.9%)	0 (0%)	2 (4.8%)
Slower side					
Contralateral	1 (5.3%)	2 (8.7%)	2 (6.9%)	1 (7.7%)	3 (7.1%)
Ipsilateral	14 (74%)	14 (61%)	16 (55%)	12 (92%)	28 (67%)
Symmetry	3 (16%)	6 (26%)	9 (31%)	0 (0%)	9 (21%)
Unclear	1 (5.3%)	1 (4.3%)	2 (6.9%)	0 (0%)	2 (4.8%)
Symmetry in amplitude					
Marked asymmetry	11 (58%)	12 (52%)	15 (52%)	8 (62%)	23 (55%)
Mild asymmetry	1 (5.3%)	3 (13%)	2 (6.9%)	2 (15%)	4 (9.5%)
Symmetry	7 (37%)	8 (35%)	12 (41%)	3 (23%)	15 (36%)
Lower amplitude side					
Contralateral	8 (42%)	12 (52%)	12 (41%)	8 (62%)	20 (48%)
Ipsilateral	4 (21%)	3 (13%)	5 (17%)	2 (15%)	7 (17%)
Symmetry	7 (37%)	8 (35%)	12 (41%)	3 (23%)	15 (36%)
Background slowing					
Absent	3 (16%)	5 (22%)	7 (24%)	1 (7.7%)	8 (19%)
Present	15 (79%)	18 (78%)	22 (76%)	11 (85%)	33 (79%)
Unclear	1 (5.3%)	0 (0%)	0 (0%)	1 (7.7%)	1 (2.4%)
Predominant background frequency on ipsilateral side					
Alpha	3 (16%)	8 (35%)	8 (28%)	3 (23%)	11 (26%)
Alpha–theta	4 (21%)	6 (26%)	9 (31%)	1 (7.7%)	10 (24%)
Delta	1 (5.3%)	1 (4.3%)	1 (3.4%)	1 (7.7%)	2 (4.8%)
Theta	1 (5.3%)	1 (4.3%)	1 (3.4%)	1 (7.7%)	2 (4.8%)
Theta–delta	9 (47%)	7 (30%)	10 (34%)	6 (46%)	16 (38%)
Unclear	1 (5.3%)	0 (0%)	0 (0%)	1 (7.7%)	1 (2.4%)
Predominant background frequency on contralateral side					
Alpha	10 (53%)	14 (61%)	19 (66%)	5 (38%)	24 (57%)
Alpha–theta	3 (16%)	4 (17%)	4 (14%)	3 (23%)	7 (17%)
Delta	0 (0%)	1 (4.3%)	0 (0%)	1 (7.7%)	1 (2.4%)
Theta	4 (21%)	1 (4.3%)	3 (10%)	2 (15%)	5 (12%)
Theta–delta	1 (5.3%)	3 (13%)	3 (10%)	1 (7.7%)	4 (9.5%)
Unclear	1 (5.3%)	0 (0%)	0 (0%)	1 (7.7%)	1 (2.4%)
Reactivity on ipsilateral side					
Absent	1 (5.3%)	3 (13%)	0 (0%)	4 (31%)	4 (9.5%)
Present	16 (84%)	19 (83%)	27 (93%)	8 (62%)	35 (83%)
Unclear	2 (11%)	1 (4.3%)	2 (6.9%)	1 (7.7%)	3 (7.1%)
Reactivity on contralateral side					
Absent	0 (0%)	1 (4.3%)	0 (0%)	1 (7.7%)	1 (2.4%)
Present	17 (89%)	21 (91%)	27 (93%)	11 (85%)	38 (90%)
Unclear	2 (11%)	1 (4.3%)	2 (6.9%)	1 (7.7%)	3 (7.1%)
At least one side with abnormal reactivity					
Absent	16 (84%)	19 (83%)	27 (93%)	8 (62%)	35 (83%)
Present	1 (5.3%)	3 (13%)	0 (0%)	4 (31%)	4 (9.5%)
Unclear	2 (11%)	1 (4.3%)	2 (6.9%)	1 (7.7%)	3 (7.1%)
Background continuity					
Continuous	19 (100%)	23 (100%)	29 (100%)	13 (100%)	42 (100%)
Ipsilateral voltage					
Low	1 (5.3%)	0 (0%)	1 (3.4%)	0 (0%)	1 (2.4%)
Normal	18 (95%)	23 (100%)	28 (97%)	13 (100%)	41 (98%)
Contralateral voltage					
Low	1 (5.3%)	0 (0%)	1 (3.4%)	0 (0%)	1 (2.4%)
Normal	18 (95%)	23 (100%)	28 (97%)	13 (100%)	41 (98%)
Ipsilateral anteroposterior gradient					
Absent	7 (37%)	9 (39%)	9 (31%)	7 (54%)	16 (38%)
Present	10 (53%)	14 (61%)	19 (66%)	5 (38%)	24 (57%)
Reverse	1 (5.3%)	0 (0%)	1 (3.4%)	0 (0%)	1 (2.4%)
Unclear	1 (5.3%)	0 (0%)	0 (0%)	1 (7.7%)	1 (2.4%)
Contralateral anteroposterior gradient					
Absent	3 (16%)	4 (17%)	5 (17%)	2 (15%)	7 (17%)
Present	15 (79%)	19 (83%)	24 (83%)	10 (77%)	34 (81%)
Unclear	1 (5.3%)	0 (0%)	0 (0%)	1 (7.7%)	1 (2.4%)
Electrographic seizures	3 (15.8%)	10 (43.5%)			13 (31%)
SEDs					
Type of SED					
Absent	7 (37%)	9 (39%)	10 (34%)	6 (46%)	16 (38%)
Sharp wave	4 (21%)	1 (4.3%)	2 (6.9%)	3 (23%)	5 (12%)
Spikes	8 (42%)	13 (57%)	17 (59%)	4 (31%)	21 (50%)
Lobar topography					
Absent	7 (37%)	9 (39%)	10 (34%)	6 (46%)	16 (38%)
Central	0 (0%)	1 (4.3%)	1 (3.4%)	0 (0%)	1 (2.4%)
Centrotemporal	1 (5.3%)	1 (4.3%)	2 (6.9%)	0 (0%)	2 (4.8%)
Frontal	0 (0%)	1 (4.3%)	0 (0%)	1 (7.7%)	1 (2.4%)
Occipital	0 (0%)	1 (4.3%)	1 (3.4%)	0 (0%)	1 (2.4%)
Temporal	10 (53%)	9 (39%)	13 (45%)	6 (46%)	19 (45%)
Unclear	1 (5.3%)	1 (4.3%)	2 (6.9%)	0 (0%)	2 (4.8%)
Dominant side					
Contralateral	2 (10.5%)	1 (4.4%)	3 (10.3%)	0 (0%)	3 (7.1%)
Ipsilateral	9 (47.4%)	12 (52.2%)	14 (48.3%)	7 (53.9%)	21 (50%)
Unclear	1 (5.3%)	1 (4.4%)	2 (6.9%)	0 (0%)	2 (4.8%)
Absent	7 (36.8%)	9 (39.1%)	10 (34.5%)	6 (46.2%)	16 (38.1%)
Prevalence of SEDs					
Absent	7 (37%)	9 (39%)	10 (34%)	6 (46%)	16 (38%)
Rare	6 (32%)	4 (17%)	8 (28%)	2 (15%)	10 (24%)
Occasional	4 (21%)	4 (17%)	7 (24%)	1 (7.7%)	8 (19%)
Frequent	2 (11%)	5 (22%)	4 (14%)	3 (23%)	7 (17%)
Abundant	0 (0%)	1 (4.3%)	0 (0%)	1 (7.7%)	1 (2.4%)
RPPs^d^	10 (53%)	12 (52%)	11 (38%)	11 (85%)	22 (52%)
Unclear	1 (5%)	0 (0%)	0 (0%)	1 (7.7%)	1 (2.4%)
Laterality					
Contralateral	1/10 (10%)	2/12 (16.7%)	1/11 (9%)	2/11 (18%)	3/22 (13.6%)
Ipsilateral	9/10 (90%)	9/12 (75%)	9/11 (82%)	9/11 (82%)	18/22 (81.8%)
Bilateral	0/10 (0%)	1/12 (8.3%)	1/11 (9%)	0/11 (0%)	1/22 (4.5%)
Localization^e^					
Central	4/10 (40%)	2/12 (16.7%)	4/11 (36.4%)	2/11 (18.2%)	6/22 (27.3%)
Temporal	9/10 (90%)	7/12 (58.3%)	7/11 (63.6%)	9/11 (81.8%)	16/22 (72.7%)
Frontal	4/10 (40%)	4/12 (33.3%)	4/11 (36.4%)	4/11 (36.4%)	8/22 (36.4%)
Occipital	4/10 (40%)	1/12 (8.3%)	5/11 (45.5%)	0/11 (.0%)	5/22 (22.7%)
Prevalence					
Rare	4/10 (40%)	3/12 (25.0%)	4/11 (36.4%)	3/11 (27.3%)	7/22 (31.8%)
Occasional	2/10 (20%)	6/12 (50.0%)	2/11 (18.2%)	6/11 (54.5%)	8/22 (36.4%)
Frequent	2/10 (20%)	2/12 (16.7%)	3/11 (27.3%)	1/11 (9.1%)	4/22 (18.2%)
Abundant	1/10 (10%)	1/12 (8.3%)	1/11 (9.1%)	1/11 (9.1%)	2/22 (9.1%)
Continuous	1/10 (10%)	0/12 (.0%)	1/11 (9.1%)	0/11 (.0%)	1/22 (4.5%)
Duration					
Very brief	0/10 (0%)	1/12 (8.3%)	0/11 (.0%)	1/11 (9.1%)	1/22 (4.5%)
Brief	5/10 (50%)	8/12 (66.7%)	7/11 (63.6%)	6/11 (54.5%)	13/22 (59.1%)
Intermediate	2/10 (20%)	2/12 (16.7%)	1/11 (9.1%)	3/11 (27.3%)	4/22 (18.2%)
Long	2/10 (20%)	1/12 (8.3%)	2/11 (18.2%)	1/11 (9.1%)	3/22 (13.6%)
Very long	1/10 (10%)	0/12 (.0%)	1/11 (9.1%)	0/11 (.0%)	1/22 (4.5%)
Distribution					
Generalized	0/10 (0%)	2/12 (16.7%)	1/11 (9.1%)	1/11 (9.1%)	2/22 (9.1%)
Lateralized	10/10 (100%)	10/12 (83.3%)	10/11 (90.9%)	10/11 (90.9%)	20/22 (90.9%)
Type					
PDs	1/10 (10%)	1/12 (8.3%)	1/11 (9.1%)	1/11 (9.1%)	2/22 (9.1%)
RDA	9/10 (90%)	11/12 (91.7%)	10/11 (90.9%)	10/11 (90.9%)	20/22 (90.9%)
Frequency					
1 Hz	2/10 (20%)	1/12 (8.3%)	2/11 (18.2%)	1/11 (9.1%)	3/22 (13.6%)
1.5 Hz	5/10 (50%)	4/12 (33.3%)	6/11 (54.5%)	3/11 (27.3%)	9/22 (40.9%)
2 Hz	2/10 (20%)	5/12 (41.7%)	2/11 (18.2%)	5/11 (45.5%)	7/22 (31.8%)
2.5 Hz	1/10 (10%)	1/12 (8.3%)	1/11 (9.1%)	1/11 (9.1%)	2/22 (9.1%)
4 Hz	0/10 (0%)	1/12 (8.3%)	0/11 (.0%)	1/11 (9.1%)	1/22 (4.5%)
Amplitude					
High	1/10 (10%)	0/12 (.0%)	1/11 (9.1%)	0/11 (.0%)	1/22 (4.5%)
Medium	9/10 (90%)	12/12 (100.0%)	10/11 (90.9%)	11/11 (100.0%)	21/22 (95.5%)
Evolution					
Evolving	3/10 (30%)	4/12 (33.3%)	2/11 (18.2%)	5/11 (45.5%)	7/22 (31.8%)
Fluctuating	3/10 (30%)	2/12 (16.7%)	4/11 (36.4%)	1/11 (9.1%)	5/22 (22.7%)
Static	4/10 (40%)	6/12 (50.0%)	5/11 (45.5%)	5/11 (45.5%)	10/22 (45.5%)
Plus features	9/10 (90%)	9/12 (75.0%)	7/11 (63.6%)	11/11 (100.0%)	18/22 (81.8%)
Plus F	1/9 (11.1%)	1/9 (11.1%)	1/7 (14.2%)	1/11 (9.1%)	2/18 (11.1%)
Plus S	8/9 (88.9%)	8/9 (88.9%)	6/7 (85.7%)	10/11 (90.9%)	16/18 (88.9%)

*Note*: Alpha, 8–12 Hz; theta, 5–7 Hz; delta, 1–4 Hz.

Abbreviations: PD, periodic discharge; Plus F, superimposed fast activity; Plus S, superimposed sharp waves or spikes, or sharply contoured; RDA, rhythmic delta activity; RPP, rhythmic and periodic pattern; SED, sporadic epileptiform discharges.

^a^ Ipsilateral and contralateral are defined in relation to the side of the hematoma.

^b^ Acute symptomatic seizure refers to seizure related to intracerebral hemorrhage, within 72 h following inclusion, including clinical and electrographic seizures. In our cohort, all seizures were electrographic.

^c^ *n* (%).

^d^ When multiple types of RPP were present, the earliest one to appear was selected.

^e^ RPPs can be present at multiple locations.

RPPs were observed in 22 patients (52%), including 90.9% patients with RDA and 9.1% patients with PDs (Table 2). RPPs were mainly ipsilateral to the hematoma (81.8%), lateralized (90.9%), and of temporal localization (72.7%). They were typically rare or occasional (68.2%) with brief duration (59.4%). Plus features were often present (81.8%), an evolving pattern was present in 31.8%, and RPP frequency ≥ 2 Hz in 45.4% of patients. Interictal EEG characteristics appeared similar between the levetiracetam and placebo groups.

#### 3.2.2 Electrographic seizures

A total of 164 ESz were recorded, with a median of 0 (Q1–Q3 = 0–2) seizures per patient (0 [Q1–Q3 = 0–6] in the placebo group versus 0 [Q1–Q3 = 0–0] in the levetiracetam group). In patients who presented seizures, the median duration of seizures was 103 (Q1–Q3 = 66–188) s (107 [Q1–Q3 = 68–191] s in the placebo group and 74 [Q1–Q3 = 35–98] s in the levetiracetam group). EEG patterns of seizures included mostly sharp waves (61.5%), with all but one seizure occurring ipsilateral to the side of the hematoma. Details of seizures are provided in Peter‐Derex et al.^16^ and in Tables S2 and S3.

### 3.3 Characteristics and temporal dynamics of RPPs according to seizures

RPPs were observed in 11 of 12 (91.7%) patients with seizures (in one patient with seizures, excessive artifacts in the recording prevented reliable RPP analysis) and in 11 of 29 (38%) patients without seizures. Characteristics of RPPs associated with seizures are summarized in Table 2. A frequency ≥ 2 Hz was observed in 63.7% of patients with seizures compared to 27.3% without. An evolving pattern was present in 45.5% of patients with seizures versus 18.2% without. All patients with seizures had RPPs with plus features, compared to 63.4% in the nonseizure group.

The median time from ICH (symptom onset) to the first RPP was 26 (Q1–Q3 = 15–40) h and to the first seizure was 49 (32–54) h. The median time from start of EEG to first RPP was .6 [.2–1.7] h.

**FIGURE 1:**
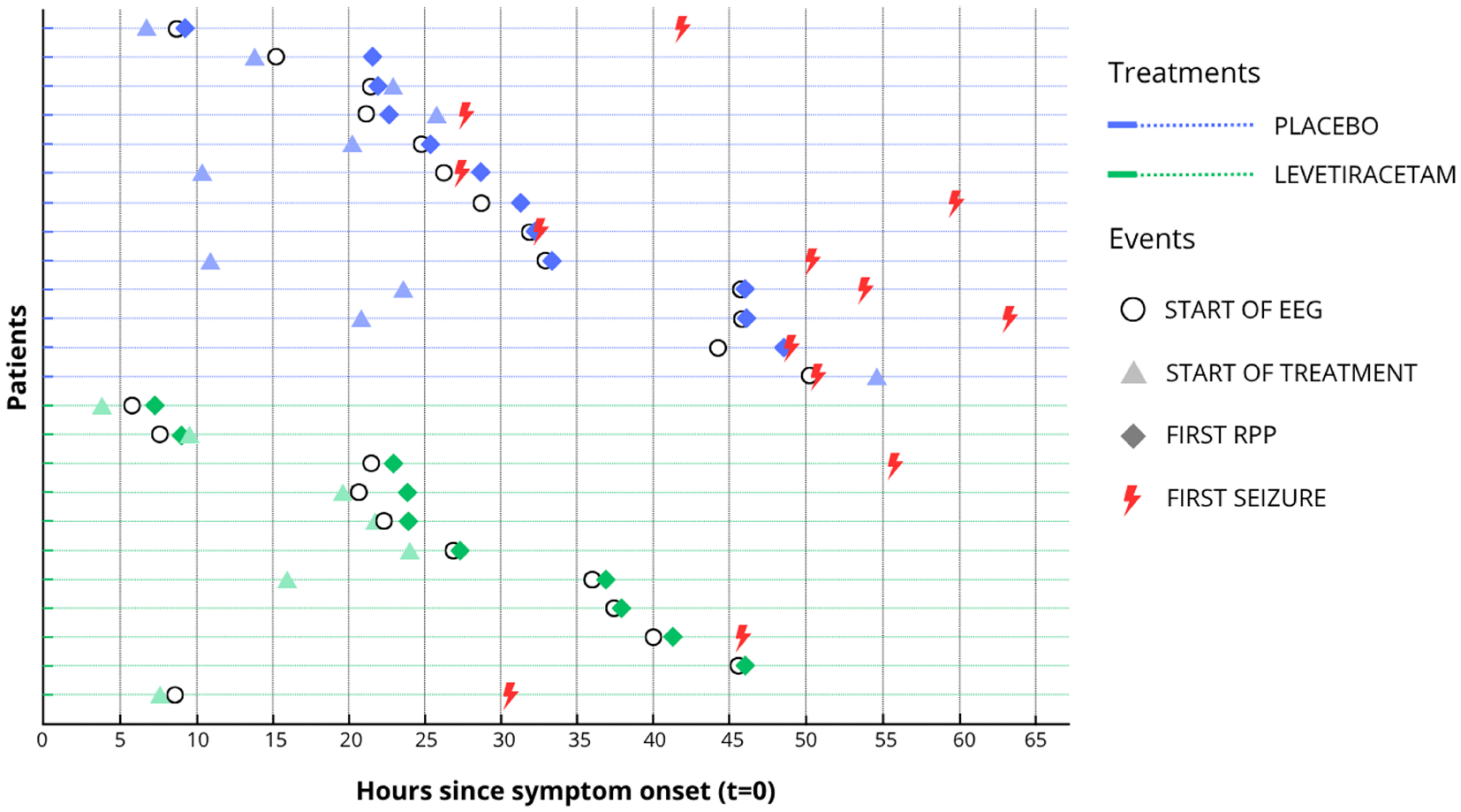
Temporal dynamics of rhythmic and periodic patterns (RPPs) and electrographic seizures. Patients are presented in descending order of first RPP timing in both the placebo and levetiracetam groups. Please note that treatment initiation timing was not available for seven patients. EEG, electroencephalogram.

Among patients with seizures in whom EEG was reliable for RPP detection (*n* = 12), RPPs preceded the first seizure in 10 cases (83.3%) and followed the first seizure in one case (8.3%), and one (8.3%) patient had seizures without any RPP during the recording. When RPPs preceded seizures, the median delay between the first RPP and seizure onset was 12 (Q1–Q3 = 4–25) h. Time from symptom onset, first RPP, and first seizure in levetiracetam and placebo groups is presented in Table 3 and Figure 1.

**TABLE 3:** Temporal dynamics of RPPs and seizures.

	Treatment		Overall, *N* = 42
	Levetiracetam, *n* = 19	Placebo, *n* = 23	
Time from ICH {symptom onset}			
To start of recording	26 [21–38]	26 [11–46]	26 [15–40]
*n*	19	23	42
To first RPP	26 [23–39]	31 [22–40]	29 [23–39]
*n*	10	12	22
To first electrographic seizure	46 [31–56]	50 [32–54]	49 [32–54]
*n*	3	10	13
Time from EEG start to first RPP	1.1 [.5–1.3]	.4 [.1–2.7]	.6 [.2–1.7]
*n*	10	12	22
RPP in patients with seizure^a^	2/2 (100%)	9/10 (90%)	11/12 (91.7%)
Seizure occurred after RPP	2/2 (100%)	8/10 (80%)	10/12 (83.3%)
Seizure occurred before RPP	0/2 (0%)	1/10 (10%)	1/12 (8.3%)
Time from RPP to first seizure^b^	18 [11–25]	12 [4–20]	12 [4–25]
*n*	2	8	10

*Note*: All time data are displayed in median hours [IQR]; other data are displayed as *n* (%).

Abbreviations: EEG, electroencephalogram; ICH, intracerebral hemorrhage; RPP, rhythmic and periodic pattern.

^**a**^ Data are missing in one patient due to excessive artifacts.

^b^ When an RPP is present and precedes seizures.

### 3.4 Factors associated with RPPs and epileptic seizures

Several radiological characteristics differed between patients with and without RPPs (Table 4). In the RPP group, median volume was higher (21 [Q1–Q3 = 10–36] cm^3^ vs. 5 [Q1–Q3 = 3–14] cm^3^, *p* = .002), and lobar localization and cortical involvement were more frequent (respectively, 45% vs. 10%, *p* = .004 and 81% vs. 21.1%, *p* < .001). Age was not associated with RPPs, nor was intraventricular extension, change in midline shift at 72 h, the presence of sporadic epileptiform discharges, or levetiracetam use (Table 4). After adjustment for multiple comparisons, only hematoma volume and cortical involvement remained significant.

**TABLE 4:** Factors associated with rhythmic and periodic patterns and seizures.

Characteristic	Rhythmic and periodic patterns				Acute symptomatic seizures^a^			
	Absent, *n* = 19^b^	Present, *n* = 22^b^	*p* ^c^	Adjusted *p* ^d^	Absent, *n* = 29^b^	Present, *n* = 13^b^	*p* ^c^	Adjusted *p* ^d^
Age, years	73 [52–80]	71 [60–78]	.8	>.9	73 [61–79]	64 [58–79]	.6	>.9
Hematoma volume, cm^3^	5 [3–14]	21 [10–36]	.**002**	.**039**	5 [3–16]	27 [20–45]	**<.001**	**<.001**
Lobar localization	1 (5.3%)	10 (45.5%)	.**004**	.053	7 (24%)	5 (38%)	.5	>.9
Cortical involvement	4 (21.1%)	17/21 (81%)	**<.001**	.**003**	10/28 (35.7%)	12 (92.3%)	**<.001**	.**01**
Change in midline shift at 72 h, cm	0 [.0–1.5]	2 [1.0–5.0]	.07	.3	0 [.0–1.0]	3 [2.0–5.0]	**<.01**	.**03**
*n*	12	13			16	9		
Sporadic epileptiform discharges	9 (47.4%)	16 (72.7%)	.1	>.9	19 (65.5%)	7 (53.3%)	.5	>.9
Levetiracetam	8 (42.1%)	10 (45.5%)	.8	>.9	16 (55.2%)	3 (23.1%)	.053	.441
Rhythmic and periodic patterns					11 (37.9%)	11/12 (91.7%)	.**002**	.**01**

*Note*: In the case of missing data, sample sizes are indicated *n*/*N*. Bold indicates significance level at *p* value < .05.

^a^ Acute symptomatic seizure refers to seizure related to intracerebral hemorrhage, within 72 h following inclusion, including clinical and electrographic seizures. In our cohort, all seizures were electrographic.

^b^ *n* (%) or median [Q1–Q3].

^c^ Wilcoxon rank‐sum test, Pearson chi‐squared test, or Fisher exact test.

^d^ Holmes correction.

Several radiological characteristics differed between patients with and without ESz (Table 4). In the seizure group, median volume was higher (27 [Q1–Q3 = 20–45] cm^3^ vs. 5 [Q1–Q3 = 3–16] cm^3^, *p* < .001), cortical involvement was more frequent (92.3% vs. 35.7%, *p* < .001), increase in midline shift at 72 h was higher (3 [Q1–Q3 = 2–5] cm vs. 0 [Q1–Q3 = 0–1] cm, *p* = .03), and prevalence of RPPs was higher (91.7% vs. 37.9%, *p* = .002). In addition, fewer patients in the seizure group were receiving levetiracetam, although the difference was not statistically significant (23.1% vs. 55.2%, *p* = .053). Notably, among patients with RPPs, seizures occurred in 75% (9/12) of cases in the placebo group versus 20% (2/10) in the levetiracetam group (*p* = .030). In contrast, hematoma lobar localization, presence of intraventricular hemorrhage, and sporadic epileptiform discharges were not significantly associated with seizures.

Examples of anatomoelectrophysiological correlations are provided in Figure S2.

**FIGURE 2:**
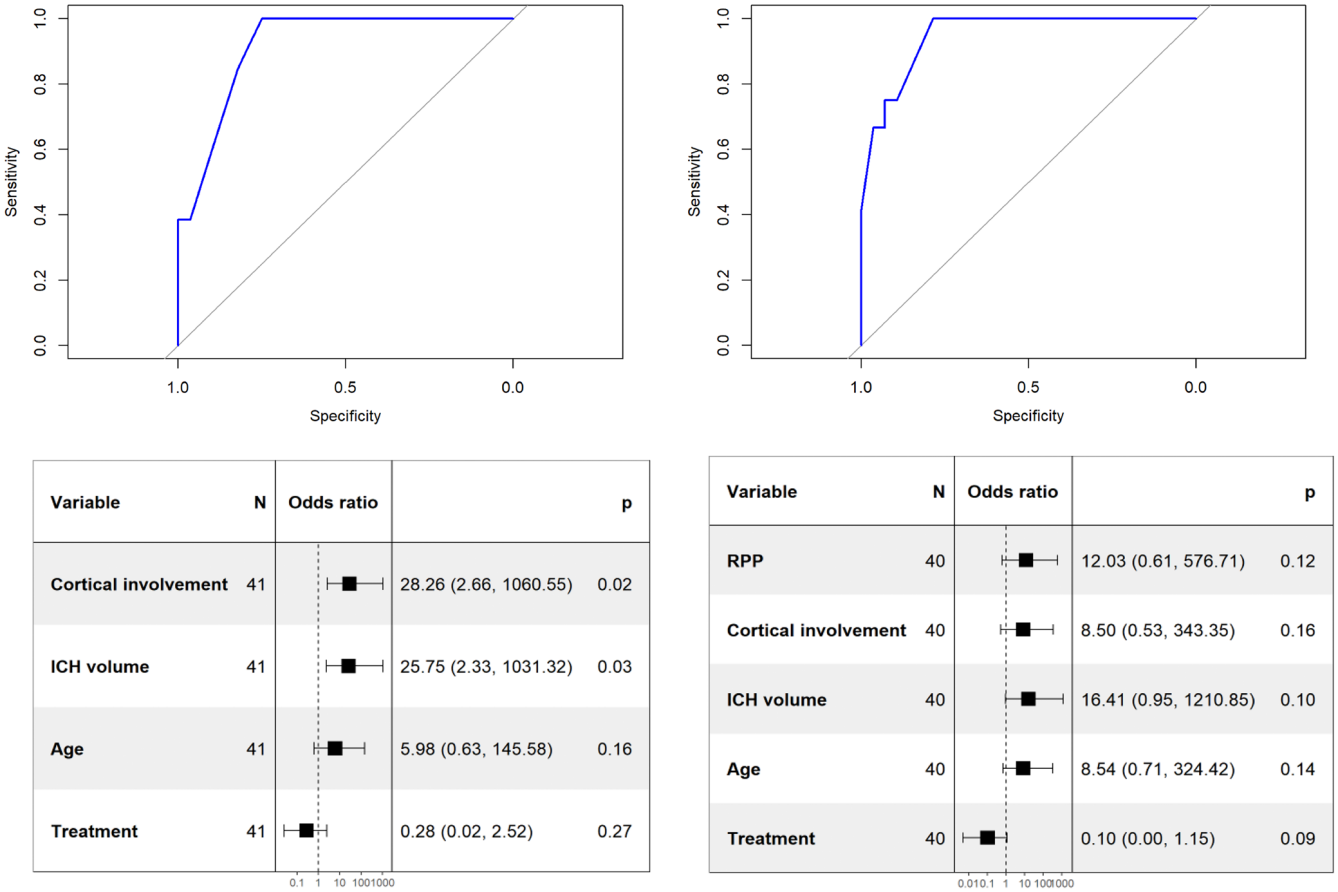
Prediction of acute symptomatic seizures. (A) receiver operating characteristic curve using cortical involvement, age < 65 years, hematoma volume > 10 mL, and levetiracetam administration to predict acute symptomatic seizures, area under the curve (AUC) = .9176. (B) Adding rhythmic and periodic patterns (RPPs) to the model, AUC = .9494. DeLong test was used for comparison, with no statistical difference (*D* = .631, *p* = .53). ICH, intracerebral hemorrhage.

Based on previous work from Bunney et al.,^26^ an exploratory analysis was conducted to test the CAV score in our population with two adapted models. Both models included cortical involvement, age < 65 years, hematoma volume > 10 mL, and levetiracetam use. The second model also included RPPs. The model including RPPs showed a slightly higher AUC (.949) compared to the model without RPPs (.918; Figure 2). However, DeLong test indicated that this difference was not statistically significant (*D* = .631, *p* = .53).

## 4 DISCUSSION

In this systematic study of EEG patterns at the acute phase of ICH, we found that (1) most (91.7%) patients with seizures have RPPs; (2) when present, RPPs precede seizures in >90% cases; (3) in patients with RPPs, the risk of seizure is reduced in patients treated with ASM; and (4) the CAV model including ASM shows good predictive performance for predicting ASyS, and the addition of RPPs may offer a modest, yet nonsignificant, improvement.

Leveraging on systematic cEEG started in the 48 h following the onset of ICH symptoms, we were able to describe EEG patterns in patients with and without ASM prophylaxis at the acute phase of brain injury. We observed several nonparoxysmal abnormalities on EEG, mostly ipsilateral to the hematoma, including slowing in the background rhythm and disruption of anteroposterior gradient, which has been associated with higher mortality in patients with ICH.^29^ An absence of reactivity was observed in 9.5% of patients, a proportion lower that the 13% reported by Claassen et al. in a retrospective study of patients with ICH and indication for cEEG.^15^ Interictal epileptiform discharges were observed in more than half of all patients, but were not associated with seizure occurrence, consistent with findings from other cEEG studies in patients with ICH.^35^ In contrast, some cEEG studies conducted in large populations of patients with diverse brain injuries—not limited to ICH—have reported an association between independent sporadic epileptiform discharges (a component of the 2HELPS2B score) and seizure occurrence, suggesting that the type of underlying brain lesion may influence the progression of paroxysmal EEG patterns.^32^

RPPs were present in half of the patients, and half of them presented seizures. We confirmed that several imaging characteristics are associated with RPPs (cortical involvement, ICH volume, lobar localization).^35^ Some of them are common to predictive factors of early seizures,^6^, ^8^, ^26^ which suggests shared pathophysiological mechanisms.^36^ RPP prevalence was similar in levetiracetam and placebo groups. However, in line with previous studies,^29^, ^31^, ^35^ we found that RPPs were associated with seizures, and in patients with RPPs, the risk of ASyS was higher in the placebo than in the levetiracetam group. This finding supports the idea that ASM would mainly act by preventing the synchronization and propagation of epileptiform discharges, rather than suppressing interictal activities.^37^, ^38^ Regardless RPP status, a strong trend toward an effect of levetiracetam on seizure occurrence was observed (*p* = .053), although it did not reach statistical significance—unlike in the PEACH trial, where the prespecified analysis was stratified by baseline NIHSS and study site.^16^ Although caution is warranted due to the small sample size, the CAV model with the addition of ASMs demonstrated good predictive performance in our population. Interestingly, incorporating EEG features (RPPs) may contribute to improving the model's performance, although limited sample size may have reduced the statistical power of the DeLong test, possibly preventing the detection of a true difference in model performance. Further studies are needed to confirm the added value of such multidimensional approaches. An original and interesting finding of our study is that RPPs occurred in most patients before the seizure, with a median delay of 35 min after EEG initiation; this suggests that a short duration early EEG may be sufficient to detect RPPs, offering a window to initiate prophylactic ASM before seizure onset. In some observational studies, early seizures after ICH have been associated with poorer functional outcomes and higher mortality.^17^, ^18^, ^19^ This deleterious effect of seizures could be explained by seizures occurring when the brain is most vulnerable. Thus, seizures have been shown to increase in intracranial pressure, potentially leading to greater midline shift^14^ as confirmed in our study; increased perihematomal edema is associated with worse functional outcomes.^39^ Another mechanism may involve impaired neurovascular coupling due to ICH‐related hypoperfusion, associated with increased metabolic demand linked to seizures.^40^, ^41^ Cortical spreading depolarizations, which have been shown to interact with epileptic activity, may also exacerbate tissue damage through metabolic stress, especially in areas of compromised perfusion.^42^, ^43^ Furthermore, the occurrence of seizures is associated with more in‐hospital complications (aspiration pneumonia), and poorer observance of antihypertensive treatment and rehabilitation due to consciousness alterations secondary to seizures. However, this poorer prognosis may also be attributable to the occurrence of seizures in patients with more severe ICH, including those with a larger ICH volume.^6^, ^24^ Thus, to date, no study has demonstrated that seizure prevention improves functional outcome and mortality, and primary ASM prophylaxis is not recommended at the acute phase of ICH.^22^, ^44^ Identifying patients at high risk of seizure might allow designing randomized clinical trials aiming to assess the potential benefit of targeted ASM prophylaxis. In addition to clinical (age) and imaging features, EEG findings may enhance risk assessment. Currently, systematic cEEG is not recommended in acute supratentorial brain injury except in cases of altered mental status.^45^ Our data highlight the potential value of early systematic EEG, despite practical challenges related to cost and organization in stroke units.

A major strength and original aspect of this study lies in the systematic early cEEG monitoring in patients with ICH. This prospective approach allowed for data collection across a broad spectrum of clinical severity and seizure risk, whereas most studies in the field have relied on retrospective analyses of intensive care unit patients undergoing cEEG due to a presumed high risk of seizures, introducing a significant selection bias.^15^, ^35^ Furthermore, treatment allocation (levetiracetam or placebo) in our study was randomized as part of the PEACH trial, rather than based on the treating physician's assessment of seizure risk.^15^ This design enabled an unbiased evaluation of treatment effects on EEG patterns. Importantly, contrary to previous studies,^14^, ^15^ physicians remained blinded to cEEG findings, ensuring that clinical management was not influenced by EEG data and allowing the natural history of EEG abnormalities to be studied. Finally, EEG interpretation adhered to the ACNS Standardized Critical Care EEG Terminology, providing a comprehensive and standardized classification of EEG patterns.^34^ However, several limitations should be acknowledged. The primary one is the small sample size, which reduced the statistical power to identify factors associated with early seizures and the robustness of our results, especially regarding the CAV model, with a potential risk of overfitting. In addition, this may have contributed to some imbalance between levetiracetam and placebo groups, despite randomization. Another limitation is the timing of cEEG monitoring, which started at a median of 26 h after symptom onset and lasted 48 h, potentially leading to missing earlier or later EEG patterns. Notably, in the only patient who experienced seizures without preceding RPPs, recording began at 50:30:10 after symptom onset and the first seizure occurred at 50:41:59, suggesting that earlier RPPs may have gone undetected. Moreover, the lack of systematic follow‐up outpatient EEGs did not allow us to assess the long‐term temporal dynamics of EEG patterns, particularly the persistence of epileptiform abnormalities. Such data would have provided valuable insights into the evolution of EEG findings and the long‐term risk of unprovoked seizures.^46^, ^47^ Finally, interrater reliability for RDA is lower than for LPDs or GPDs, which is a limitation given that RDA was the most prevalent pattern in our study.^48^

## 5 CONCLUSIONS

Our findings underscore the strong predictive value of RPPs for early seizures in patients with acute ICH. As they precede seizures in >90% of cases, RPPs emerge as an early marker of ictogenesis and may offer a critical therapeutic window for personalized prophylactic antiseizure medication, thus supporting the use of systematic cEEG monitoring in ICH.

## AUTHOR CONTRIBUTIONS

Laure Peter‐Derex and Laurent Derex were the coordinating investigators who oversaw all study conduct. Laure Peter‐Derex, Laurent Derex, Julie Haesebaert, and Sylvain Rheims contributed to the study design and data interpretation. Laurent Derex, Frédéric Philipeau, Pierre Garnier, Nathalie Andre‐Obadia, Sébastien Boulogne, Hélène Catenoix, Laure Mazzola, Julia Fontaine, Laura Mechtouff, Elodie Ong, Tae‐Hee Cho, Yves Berthezene, Nathalie Perreton, and Anne Termoz participated in study conduct and data collection. Ziad Al‐Fatuhi‐Al‐Jundi, Salomé Avenas, Muriel Rabilloud, Pierre Tankéré, Laure Peter‐Derex, and Laurent Derex contributed to data analysis. Ziad Al‐Fatuhi‐Al‐Jundi, Salomé Avenas, Muriel Rabilloud, Pierre Tankéré, Laure Peter‐Derex, Laurent Derex, and Sylvain Rheims contributed to drafting the manuscript. All authors reviewed, contributed to, and approved the final manuscript.

## FUNDING INFORMATION

This work was supported by the French Ministry of Health (PHRC‐I grant, 2013).

## CONFLICT OF INTEREST STATEMENT

L.P.‐D. reports speaker's honoraria and travel fees from Bioprojet, Jazz Pharmaceuticals, Roche, and UCB. P.T. reports speaker's honoraria and travel fees from Asdia, Resmed, Linde, Agiradom and ALLP; grant support through his institution from the “Agir pour les maladies chroniques” foundation, Bioprojet, and ALLP. S.B. reports speaker's honoraria and travel fees from Esai, LivaNova, UCB, and Angelini Pharma. S.R. reports speaker or consultant fees from Angelini Pharma, Eisai, Jazz Pharmaceuticals, LivaNova, Neuraxpharm, and UCB Pharma. L.D. reports speaker's honoraria and travel fees from Alexion, Boehringer Ingelheim, Pfizer, and Servier. L.Me. reports speaker's honoraria and travel fees from Amgen, Sanofi, and Novartis. None of the other authors has any conflict of interest to disclose. We confirm that we have read the Journal's position on issues involved in ethical publication and affirm that this report is consistent with those guidelines.

## Supporting information


**Table S1.** Framework of electroencephalographic description according to American Clinical Neurophysiology Society guidelines and terminology.
**Table S2.** Electrographic description of seizures.
**Table S3.** Electrographic description of the first seizure for every patient.
**Figure S1.** Flowchart of patient inclusion.
**Figure S2.** Examples of anatomoelectrophysiological correlations.

## Data Availability

The data that support the findings of this study are available from the corresponding author upon reasonable request and in compliance with local ethical regulations.
