# Supplementary material for: Dynamics of early electroencephalographic patterns and epileptic seizures in acute intracerebral hemorrhage: A prospective controlled study
Source: Epilepsia. 2025 Nov 18;67(3):1433–48. doi: 10.1111/epi.70017 (PMC13007832; doi:10.1111/epi.70017)

<u>**Supplementary material of the article**</u>

Dynamics of early electroencephalographic patterns and epileptic seizures in acute intracerebral hemorrhage: a prospective controlled study

Ziad Al-Fatuhi-Al-Jundi[1], Salomé Avenas[2], Pierre Tankéré[3], Frédéric Philipeau[4], Pierre Garnier[5], Laure Mazzola[6], Nathalie Andre-Obadia[1,7], Sébastien Boulogne[1,7], Hélène Catenoix[1,7], Sylvain Rheims[1,7], Tae-Hee Cho[8], Julia Fontaine[8], Laura Mechtouff[8], Elodie Ong[8], Yves Berthezene[9], Anne Termoz[10,11], Nathalie Perreton[10], Julie Haesebaert[10,11], Muriel Rabilloud[2,12], Laurent Derex[8,11], Laure Peter-Derex[3,7].

[1] Department of Functional Neurology and Epileptology, Neurological Hospital, University Hospital, Lyon, France
[2] Department of Biostatistics, Edouard Herriot Hospital, Lyon University Hospital, Lyon, France
[3] Centre for Sleep Medicine, Croix-Rousse Hospital, University Hospital, Lyon, France
[4] Stroke Unit, Department of Neurology, Fleyriat Hospital, Bourg en Bresse, France
[5] Stroke Centre, Department of Neurology, Saint-Etienne University Hospital, Saint-Etienne, France
[6] Clinical Neurophysiology Unit, Department of Neurology, Saint-Etienne University Hospital, Saint-Etienne, France
[7] Lyon Neuroscience Research Centre, CNRS UMR 5292, INSERM U1028, Lyon, France
[8] Stroke Centre, Neurological Hospital, University Hospital, Lyon, France
[9] Department of Neuroradiology, Neurological Hospital, University Hospital, Lyon, France
[10] Public Health Unit, Clinical Research and Epidemiology Department, Lyon University Hospital, Lyon, France
[11] Research on Healthcare Performance RESHAPE, INSERM U1290, Université Claude Bernard Lyon 1, France
[12] Biometry and Evolutionary Biology Laboratory, CNRS UMR 5558, Biostatistics Health Team, Villeurbanne, France

Correspondence to: ziad.Al-Fatuhi-Al-Jundi@chu-lyon.fr; laure.peter-derex@chu-lyon.fr
Centre for Sleep Medicine, Croix-Rousse Hospital, University Hospital, Lyon, France

Table S1 : Framework of EEG description according to American Clinical Neurophysiology Society guidelines and terminology [1]

[1]. Hirsch LJ, Fong MWK, Leitinger M, et al. American Clinical Neurophysiology Society's Standardized Critical Care EEG Terminology: 2021 Version. Journal of clinical neurophysiology : official publication of the American Electroencephalographic Society. 2021 ***38:*** *1-29.*

Background rhythm	-frequency or amplitude asymmetry,<br><br>-predominant frequency during wake state on each hemisphere,<br><br>-continuity,<br><br>-voltage,<br><br>-presence of an anteroposterior gradient on each hemisphere
Sporadic epileptiform discharges	-type: spikes/polyspikes/sharp wave,<br><br>-lobar topography,<br><br>-dominant side,<br><br>-prevalence
Rhythmic and periodic patterns (RPP), consisting of<br>1)periodic discharges (PDs)= repetition of a waveform (lasting <0.5 seconds, regardless of number of phases, or waveforms ≥0.5 seconds with no more than 3 phases) with relatively uniform morphology and duration, and with a clearly discernible interdischarge interval between consecutive waveforms and recurrence of the waveform at nearly regular intervals (varying by <50% from one cycle to the next in most (>50%) cycle pairs)<br>2)rhythmic delta activity (RDA)= repetition of a waveform with relatively uniform morphology and duration, without an interval between consecutive waveforms and with an activity from 0.5 to ≤4.0 Hz. A pattern can qualify as	-distribution(Generalized/Lateralized/Bilateral independent or Unilateral Independent),<br><br>-localization,<br><br>-side,<br><br>-prevalence (Continuous ≥90%, Abundant 50-89%, Frequent 10-49%, Occasional 1-9%, Rare <1%),<br><br>-duration (very long <1h, long 10-59 min, Intermediate 1-9.9 min, Brief 10-59 s, very brief <10 s),<br><br>-frequency (0-4Hz),<br><br>-Sharpness for PDs,<br><br>-Absolute Amplitude (High ≥150 µV, Medium 50-149 µV, Low 20-49 µV, Very Low <20 µV),<br><br>-evolution (Evolving in frequency, morphology and/or location; Fluctuating; or Static).

rhythmic or periodic if and only if it continues for at least 6 cycles.	-“Plus features” such as superimposed fast activity, superimposed rhythmic or quasi-rhythmic delta activity (can be applied to PDs only), with associated sharp waves or spikes, or sharply contoured (can be applied to RDA only).
Electrographic Seizure (ESz)	- starting side,<br>- lobar topography,<br>- time of onset and end,<br>- type of discharge (Spikes, Sharp Wave, Slow Wave).

Table S2: Electrographic description of seizures

† n (%), median [IQR]

††For some patients, low-voltage fast activity was visible prior to the emergence of spikes, sharp waves, or slow waves

Seizure characteristic	Levetiracetam N = 19 †	Placebo N = 23 †	Overall N= 42 †
**Number of seizures per patient**	0 [0-0]	0 [0-6]	0 [0-2]
**Patients with seizures**	3	10	13
**Number of seizures in patient with seizures**	2 [1.5-2.5]	7.5 [2.3-9.8]	3 [2-9]
**First seizure onset side**			
Ipsilateral	2/3 (66.7%)	10/10 (100%)	12/13 (92.3%)
Contralateral	1/3 (33.3%)	0/10 (0%)	1/13 (7.7%)
**First seizure type of discharge** ††			
Spikes	1/3 (33.3%)	2/10 (20%)	3/13 (23.1%)
Sharp waves	2/3 (66.7%)	6/10 (60%)	8/13 (61.5%)
Slow waves	0/3 (0%)	2/10 (20%)	2/13 (15.4%)
**Duration of seizures (sec)**	74 [35-98]	107 [68-191]	103 [66-188]
**n**	3	10	13

Table S3: Electrographic description of the first seizure for every patient

†† For some patients, low-voltage fast activity was visible prior to the emergence of spikes, sharp waves, or slow waves

Patient	*Treatment*	*Starting Side*	*Starting Lobar topography*	*Type of discharge*††	*Duration of the seizure*	**Total *Number of seizures***
1 002	PLACEBO	Ipsilateral	Temporal	Sharp Waves	0:01:14	3
1 003	PLACEBO	Ipsilateral	Occipital	Slow Waves	0:03:53	6
1 004	PLACEBO	Ipsilateral	Central	Sharp Waves	0:00:50	10
1 005	LEVETIRACETAM	Ipsilateral	Centro-temporal	Spikes	0:02:52	3
4 006	LEVETIRACETAM	Contralateral	Temporal	Sharp Waves	0:00:27	2
1 011	PLACEBO	Ipsilateral	Temporal	Sharp Waves	0:00:20	2
3 015	PLACEBO	Ipsilateral	Centro-temporal	Spikes	0:07:23	62
3 018	PLACEBO	Ipsilateral	Temporal	Spikes	0:03:59	2
1 020	PLACEBO	Ipsilateral	Temporal	Sharp Waves	0:01:47	53
1 023	LEVETIRACETAM	Ipsilateral	Frontal	Sharp Waves	0:00:58	1
1 028	PLACEBO	Ipsilateral	Frontal	Slow Waves	0:00:08	2
1 032	PLACEBO	Ipsilateral	Temporal	Sharp Waves	0:02:58	9
1 049	PLACEBO	Ipsilateral	Temporal	Sharp Waves	0:01:29	9

Figure S1. Flow chart of patients inclusion

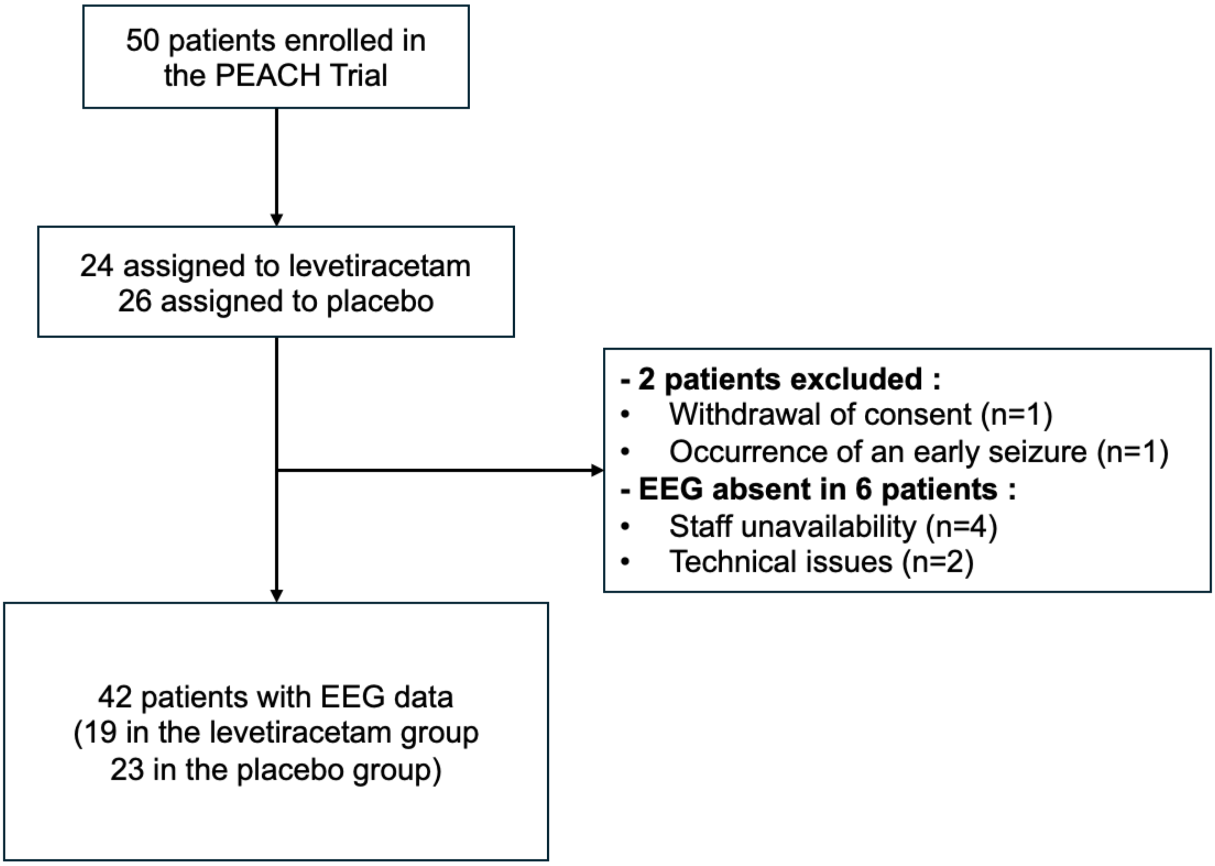

Figure S2. Examples of anatomo-electrophysiological correlations

Background Slowing: --------------
Spikes: ↙
Rhythmic delta activity: [yellow box]
Start of seizure: ⬇ (red)
End of seizure: ⬆ (red)

Patient n°12	
**ICH localization**	Deep
**ICH Volume**	4 mL
**Cortical involvement**	No
**RPP**	No
**Seizures**	No
**Treatment allocation**	Placebo

Baseline MRI (left, FLAIR; right: T2*)

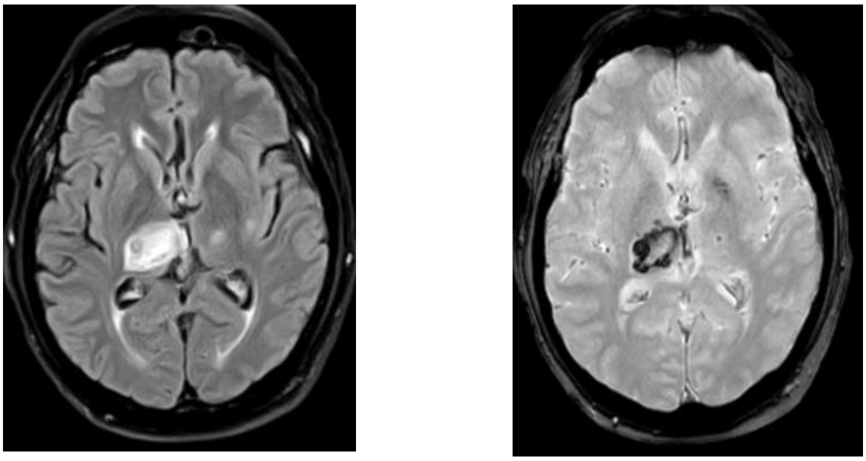

Background EEG rhythm (wake state)
Symmetric alpha rhythm (8–12 Hz) with preserved anteroposterior gradient

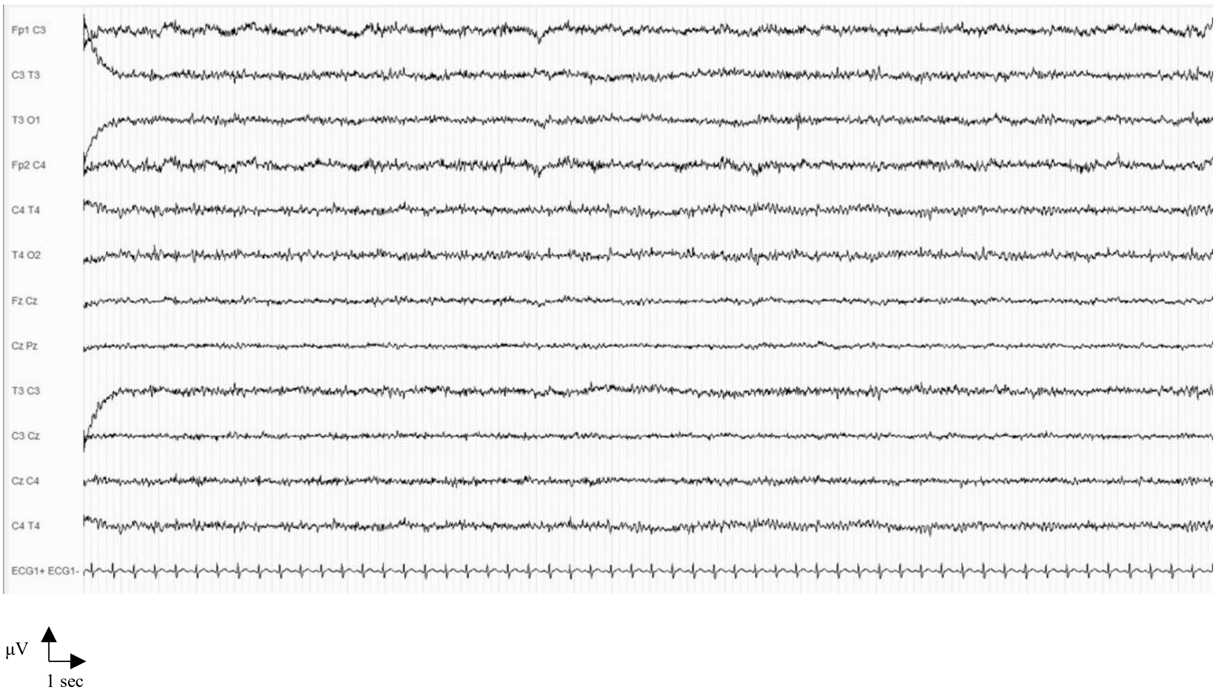

Patient n°5	
**ICH localization**	Lobar
**ICH Volume**	64 mL
**Cortical involvement**	Yes
**RPP**	Yes
**Seizures**	Yes
**Treatment allocation**	Levetiracetam

Baseline MRI (left, FLAIR; right: T2*)

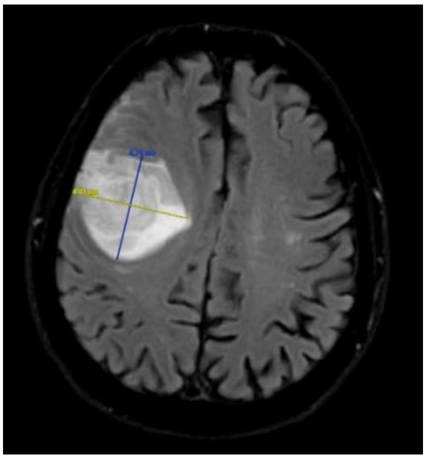

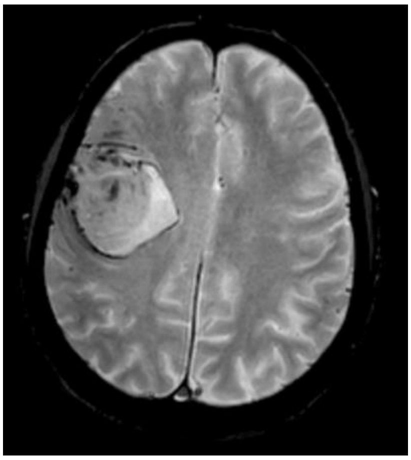

Background EEG rhythm (wake state)

Asymmetric theta activity with preserved anteroposterior gradient and right temporal delta slowing

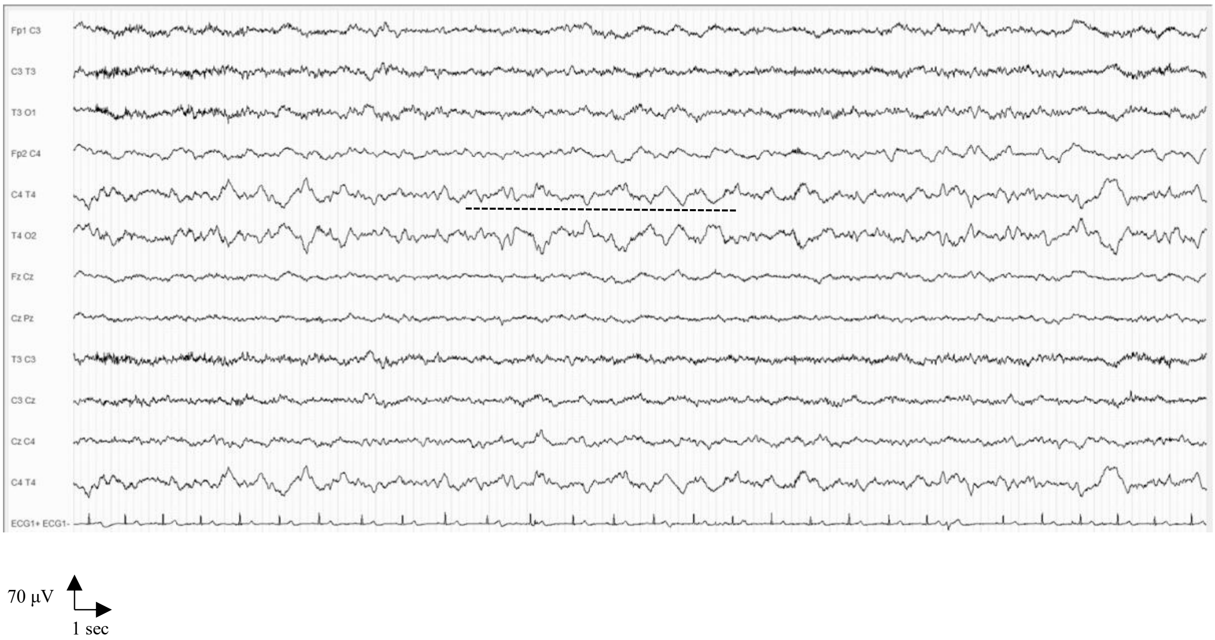

Right Temporal Rhythmic Delta Activity:

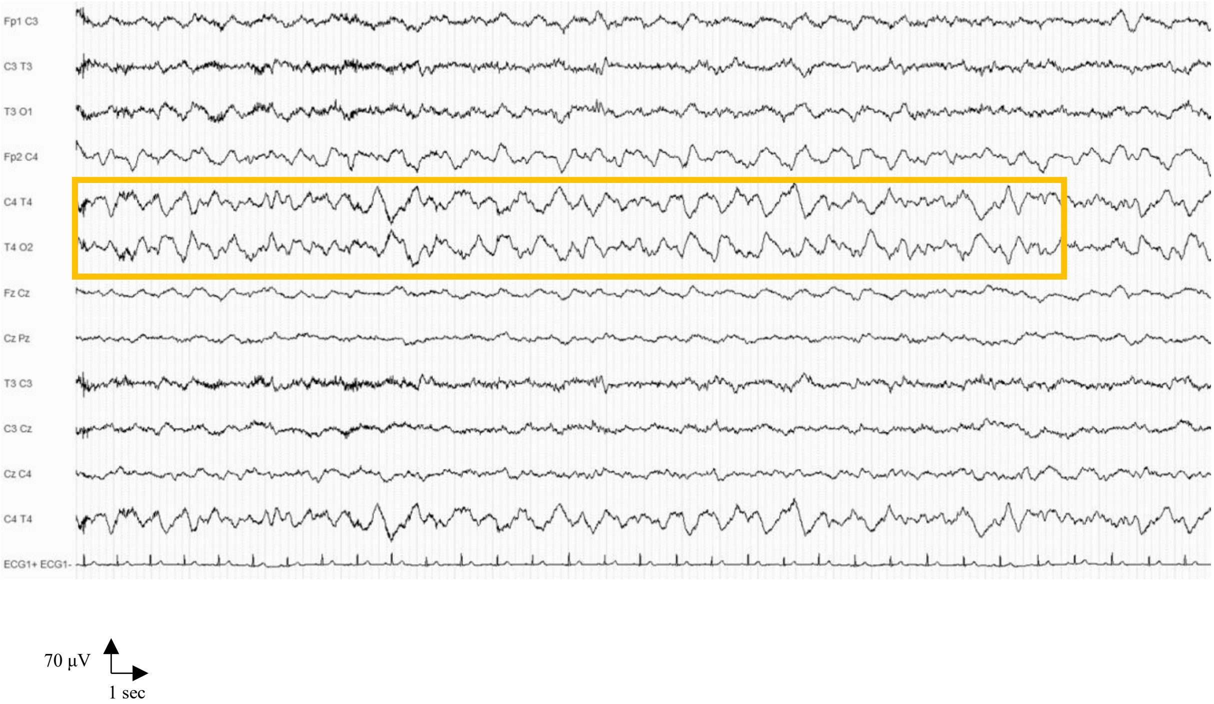

Right temporal electrographic seizure

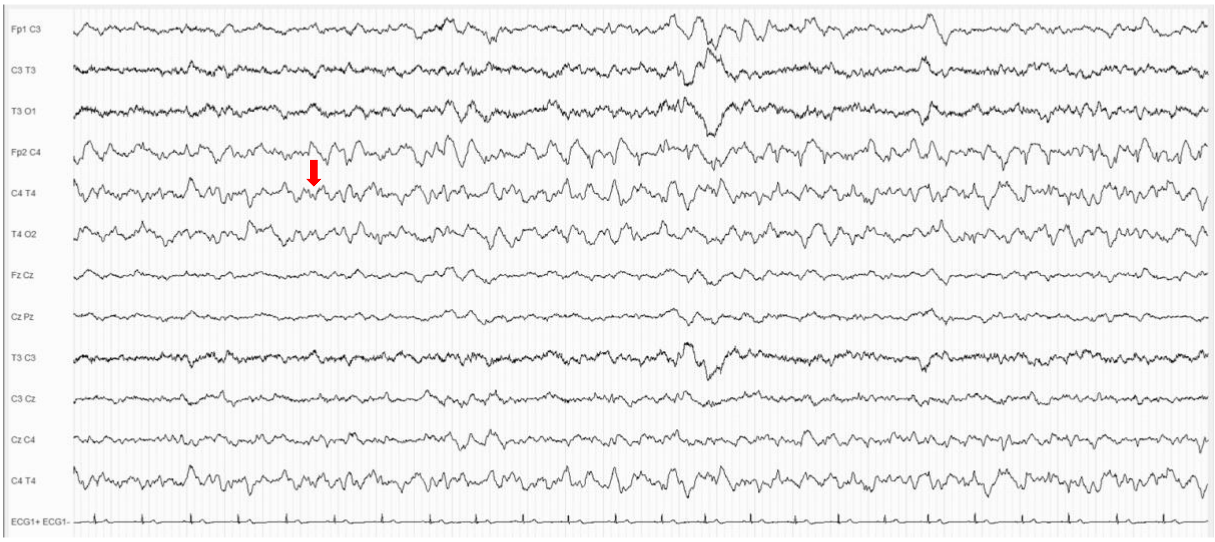

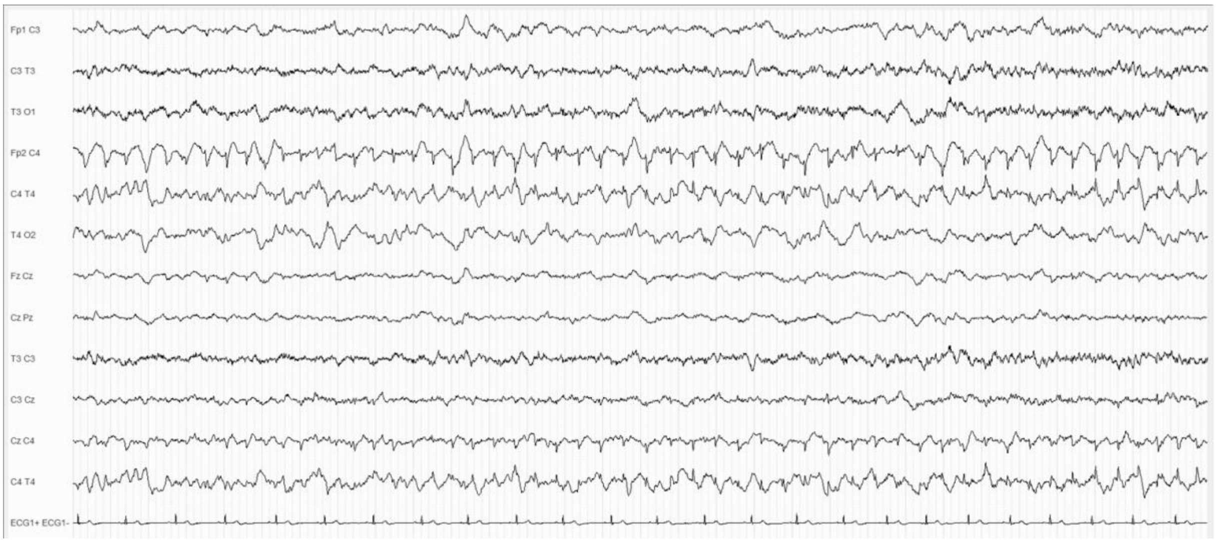
Fp1 C3
C3 T3
T3 O1
Fp2 C4
C4 T4
T4 O2
Fz Cz
Cz Pz
T3 C3
C3 Cz
Cz C4
C4 T4
ECG1+ ECG1-

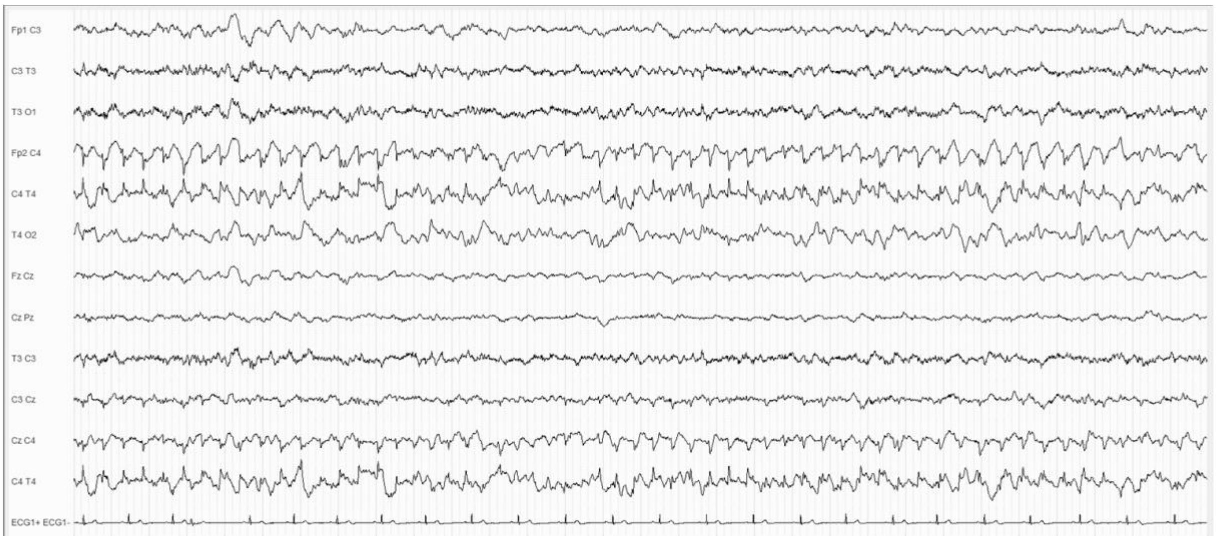
Fp1 C3
C3 T3
T3 O1
Fp2 C4
C4 T4
T4 O2
Fz Cz
Cz Pz
T3 C3
C3 Cz
Cz C4
C4 T4
ECG1+ ECG1-

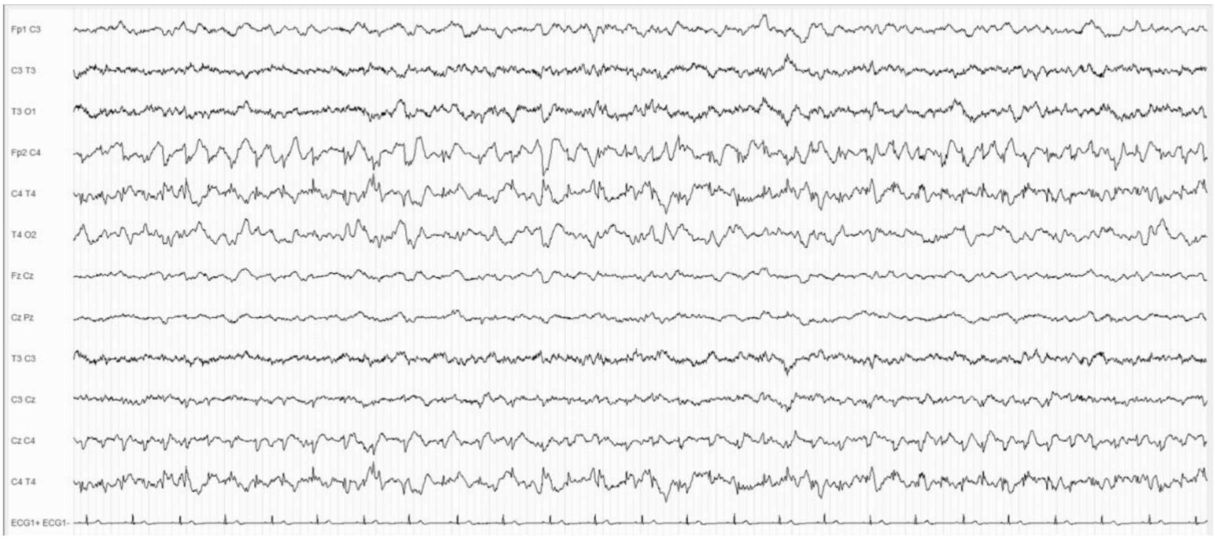
Fp1 C3
C3 T3
T3 O1
Fp2 C4
C4 T4
T4 O2
Fz Cz
Cz Pz
T3 C3
C3 Cz
Cz C4
C4 T4
ECG1+ ECG1-

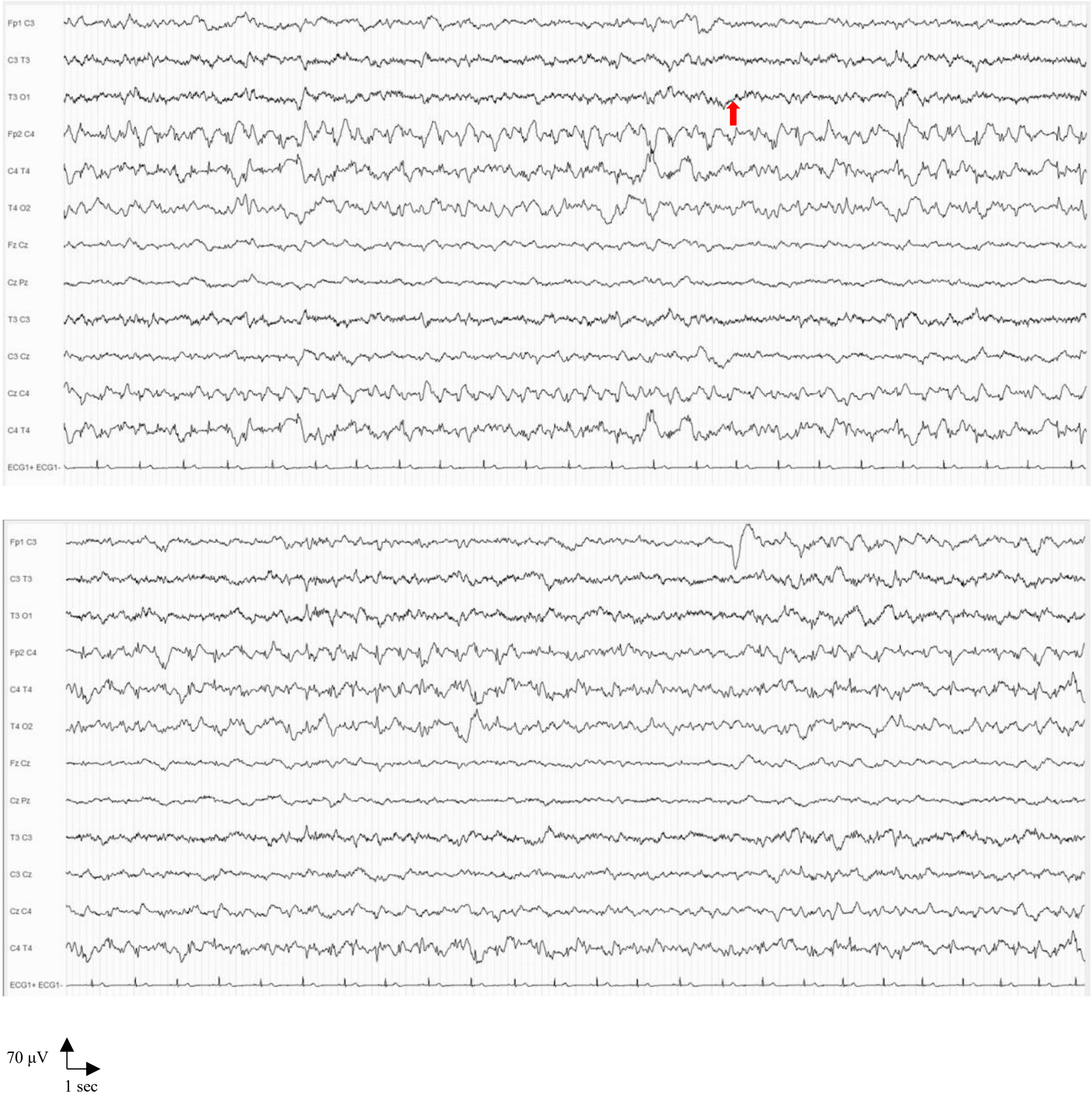
Fp1 C3
C3 T3
T3 O1
Fp2 C4
C4 T4
T4 O2
Fz Cz
Cz Pz
T3 C3
C3 Cz
Cz C4
C4 T4
ECG1+ ECG1-
Fp1 C3
C3 T3
T3 O1
Fp2 C4
C4 T4
T4 O2
Fz Cz
Cz Pz
T3 C3
C3 Cz
Cz C4
C4 T4
ECG1+ ECG1-
70 µV
1 sec

**Patient n°20**	
**ICH localization**	Deep
**ICH Volume**	65 mL
**Cortical involvement**	Yes
**RPP**	Yes
**Seizures**	Yes
**Treatment allocation**	Placebo

Baseline MRI (left, FLAIR; right: T2*)

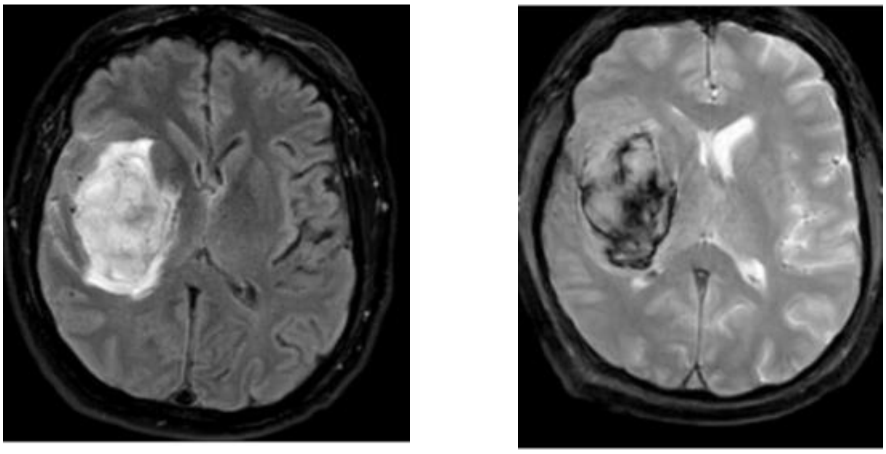

Background EEG rhythm (wake state)

Asymmetric background rhythm, with left alpha rhythm associated with a loss of anteroposterior gradient and a right hemispheric focal delta slowing with spikes.

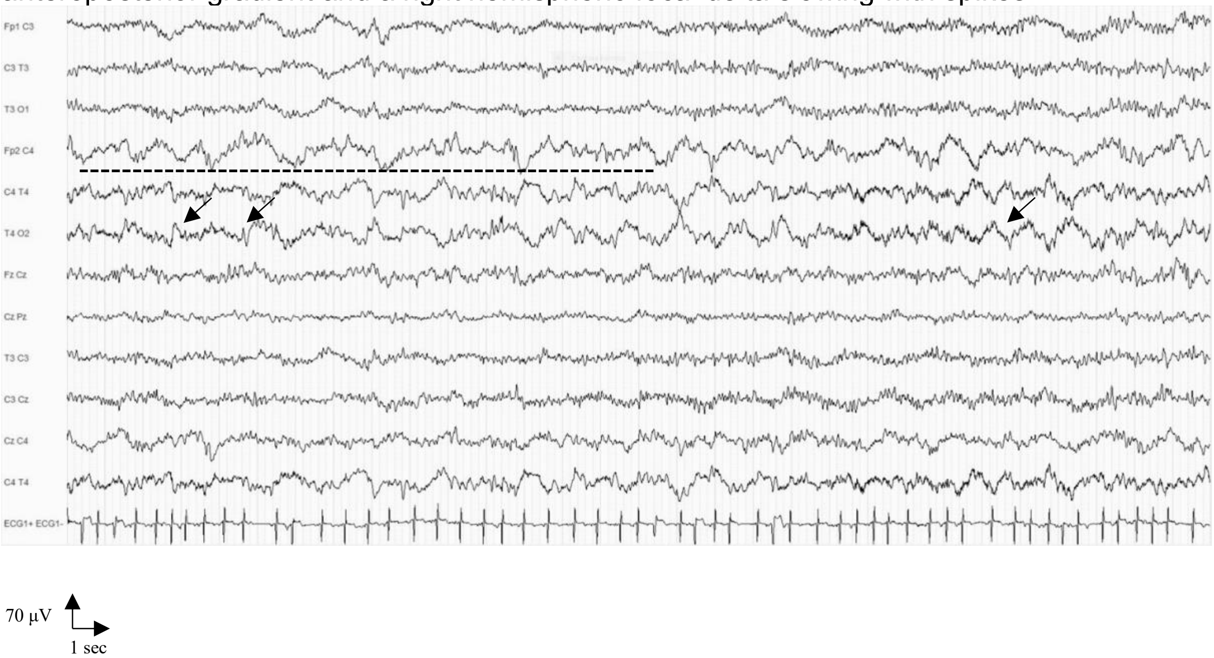

Fluctuating Rhythmic Delta Activity with Superimposed sharp waves

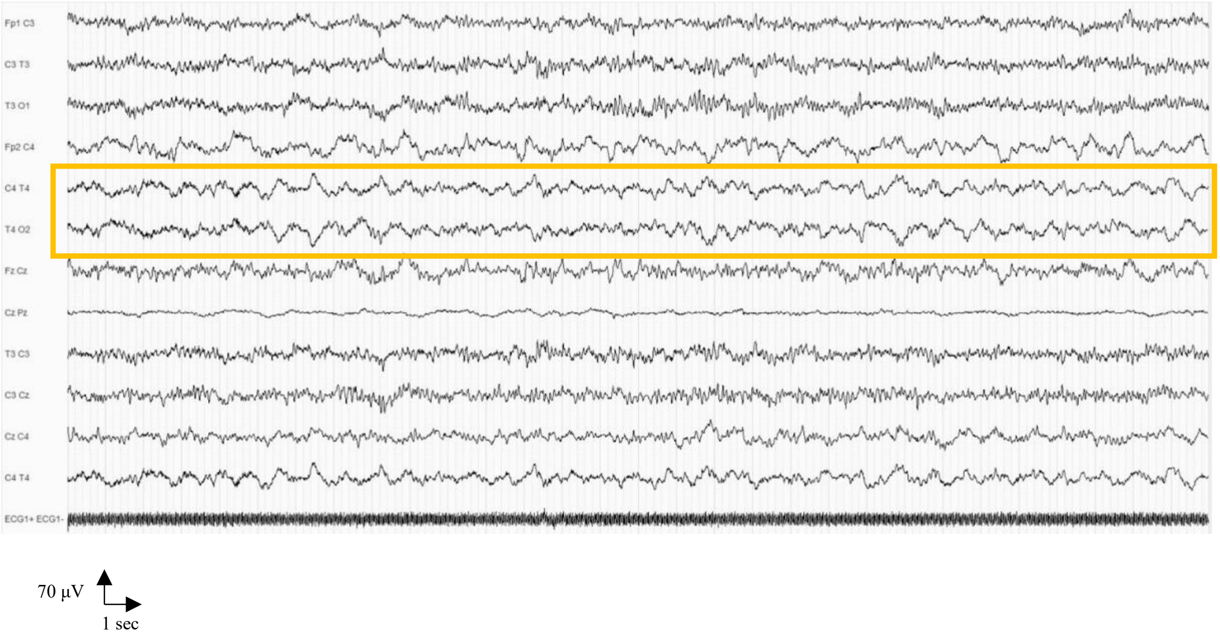

Right temporal electrographic seizure

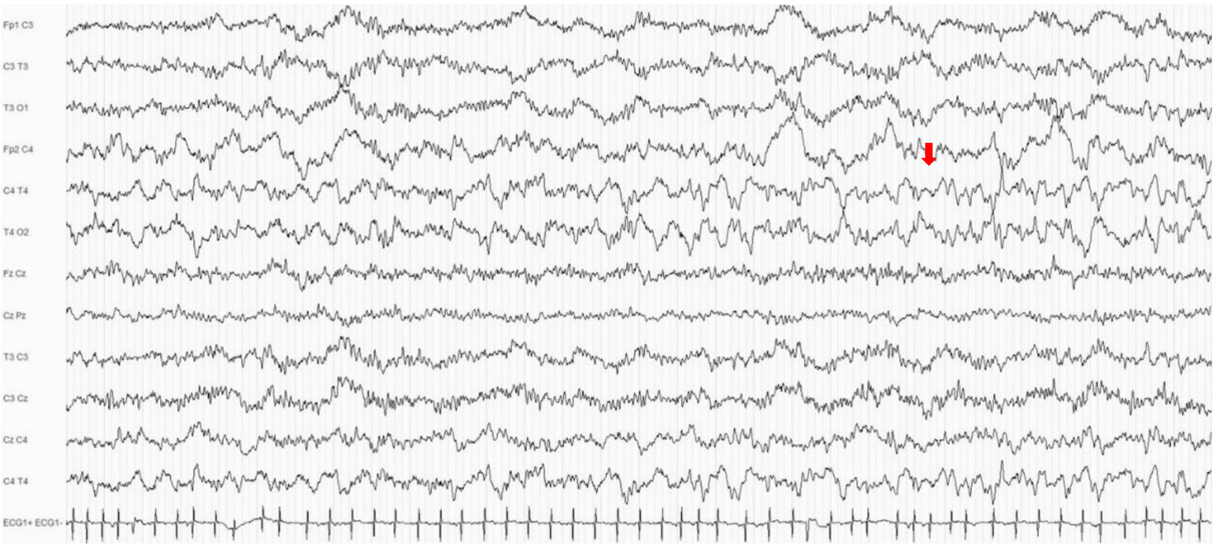

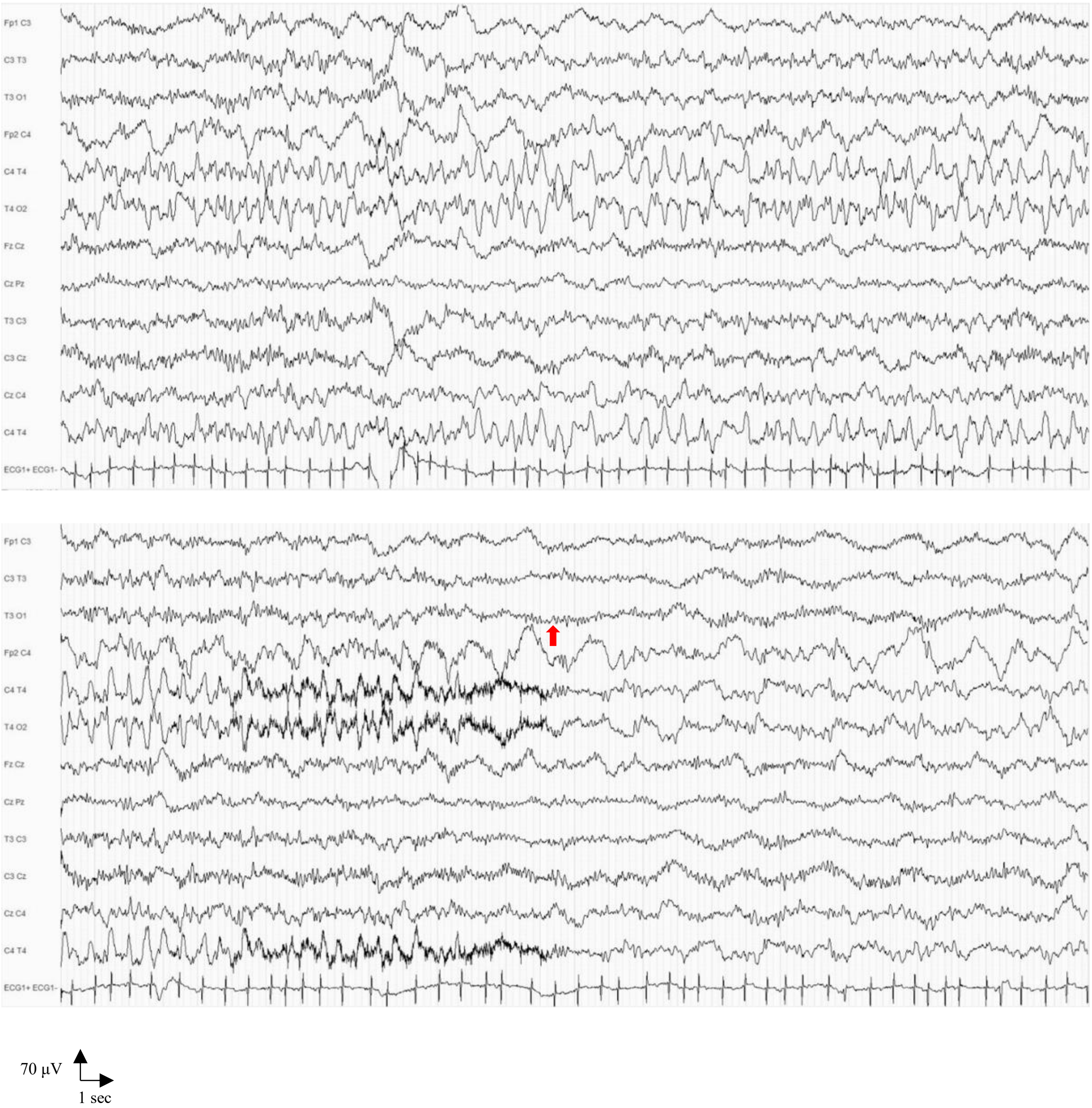
Fp1 C3
C3 T3
T3 O1
Fp2 C4
C4 T4
T4 O2
Fz Cz
Cz Pz
T3 C3
C3 Cz
Cz C4
C4 T4
ECG1+ ECG1-
Fp1 C3
C3 T3
T3 O1
Fp2 C4
C4 T4
T4 O2
Fz Cz
Cz Pz
T3 C3
C3 Cz
Cz C4
C4 T4
ECG1+ ECG1-
70 μV
1 sec

**Patient n°10**	
**ICH localization**	Lobar
**ICH Volume**	16 mL
**Cortical involvement**	Yes
**RPP**	Yes
**Seizures**	No
**Treatment allocation**	Levetiracetam

Baseline CT scan

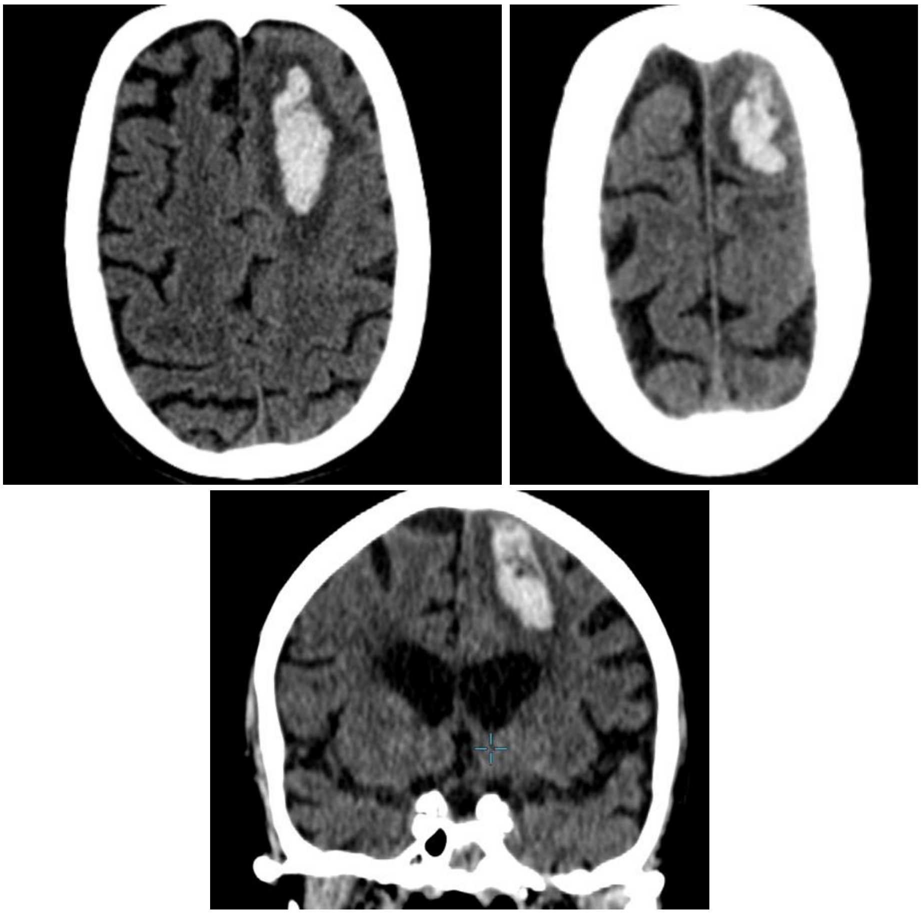

Background EEG rhythm (wake state)

Asymmetric background rhythm with right alpha rhythm associated with a preserved anteroposterior gradient and a left posterior alpha rhythm with frontal theta slowing.

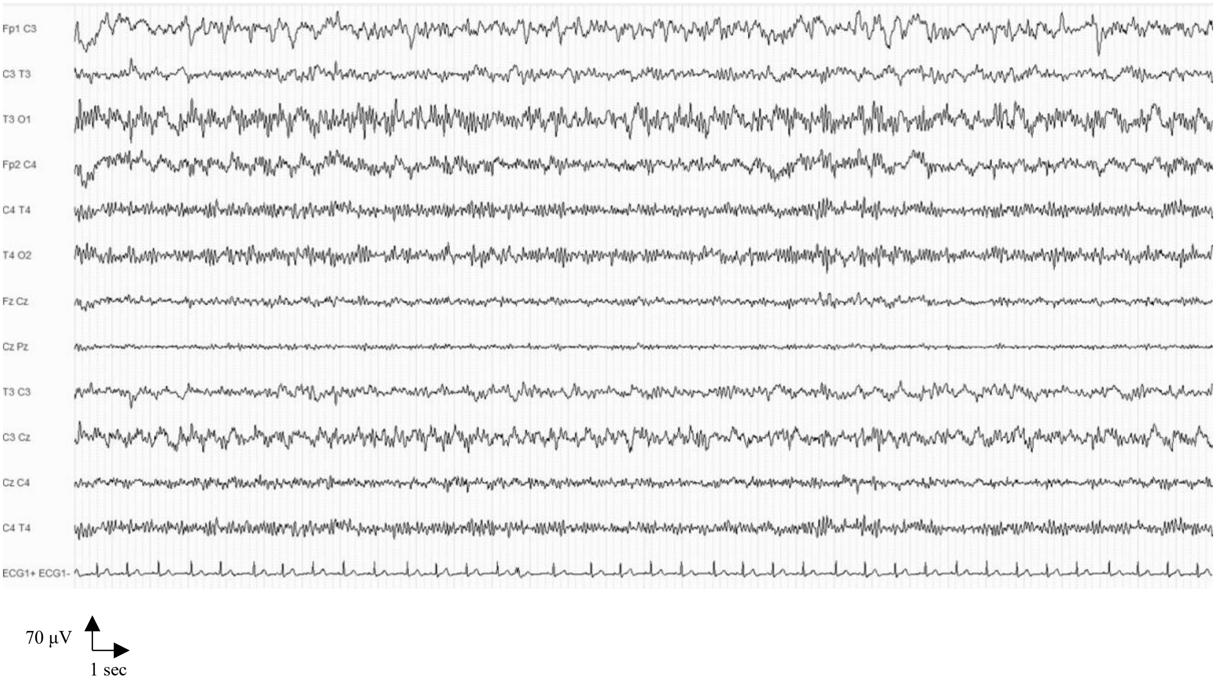

Rythmic left frontal activity

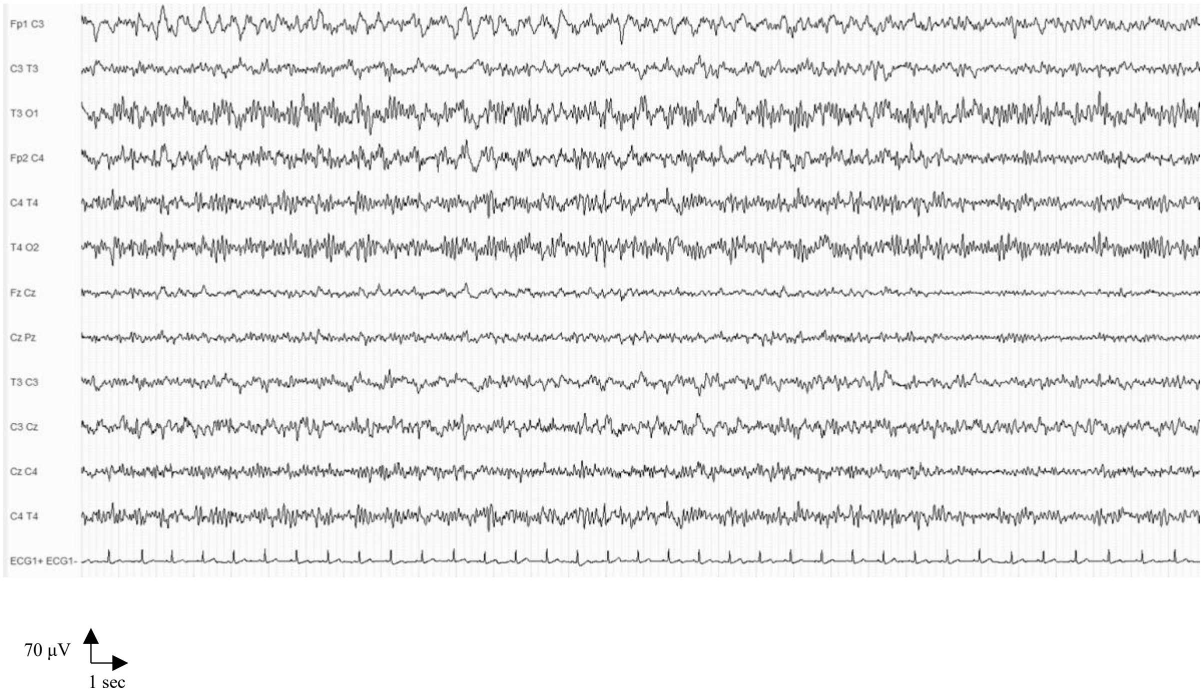

Supplement: Supplementary file 1 — Table S1. Framework of electroencephalographic description according to American Clinical Neurophysiology Society guidelines and terminology. Table S2. Electrographic description of seizures. Table S3. Electrographic description of the first seizure for every patient. Figure S1. Flowchart of patient inclusion. Figure S2. Examples of anatomoelectrophysiological correlations. [file EPI-67-1433-s001.pdf]
